# Synergistic gene expression during the acute phase response is characterized by transcription factor assisted loading

**DOI:** 10.1038/s41467-017-02055-5

**Published:** 2017-11-29

**Authors:** Ido Goldstein, Ville Paakinaho, Songjoon Baek, Myong-Hee Sung, Gordon L. Hager

**Affiliations:** 1 Laboratory of Receptor Biology and Gene Expression, CCR, NCI, NIH, Bethesda, MD 20892 USA; 2 Laboratory of Molecular Biology and Immunology, NIA, NIH, Baltimore, MD 21224 USA

## Abstract

The cytokines interleukin 1β and 6 (IL-1β, IL-6) mediate the acute phase response (APR). In liver, they regulate the secretion of acute phase proteins. Using RNA-seq in primary hepatocytes, we show that these cytokines regulate transcription in a bifurcated manner, leading to both synergistic and antagonistic gene expression. By mapping changes in enhancer landscape and transcription factor occupancy (using ChIP-seq), we show that synergistic gene induction is achieved by assisted loading of STAT3 on chromatin by NF-κB. With IL-6 treatment alone, STAT3 does not efficiently bind 20% of its coordinated binding sites. In the presence of IL-1β, NF-κB is activated, binds a subset of enhancers and primes their activity, as evidenced by increasing H3K27ac. This facilitates STAT3 binding and synergistic gene expression. Our findings reveal an enhancer-specific crosstalk whereby NF-κB enables STAT3 binding at some enhancers while perturbing it at others. This model reconciles seemingly contradictory reports of NF-κB-STAT3 crosstalk.

## Abstract (web summary)

The cytokines IL-1β and IL-6 mediate the systemic acute phase response (APR). Here, the authors provide evidence that these cytokines lead to both synergistic and antagonistic gene expression during APR; synergistic induction occurs by assisted loading of STAT3 on chromatin by NF-κB.

## Introduction

The systemic inflammatory response to pathogen infection, local tissue injury or other trauma is mediated by pro-inflammatory cytokines, mainly interleukin 1β (IL-1β), interleukin-6 (IL-6) and tumor necrosis factor (TNF). These cytokines initiate the acute phase response (APR) which is characterized by fever, changes in hormone secretion and increased white blood cell production^1^. The liver plays a major role in this systemic response by production of acute phase proteins (APP), defined as plasma proteins whose expression significantly changes during inflammation. APPs are thought to curtail pathogens and minimize tissue damage by several ways including inhibition of bacterial proteinases, modulation of iron homeostasis, increased activity of the complement system and elevation of pathogen recognition receptors^1–4^. Although mostly associated with acute inflammation and bacterial infection, IL-1β, IL-6 and TNF signaling pathways in the liver also play a significant role in disorders involving chronic inflammation such as obesity, insulin resistance, non-alcoholic steatohepatitis (NASH), viral hepatitis and fibrosis^5–7^.

The increase in hepatocyte-produced APP plasma levels can reach up to 30,000 fold in some cases^1^. Such a massive increase is heavily reliant on transcriptional regulation mediated by pro-inflammatory cytokines. Upon binding to the IL-6 receptor, IL-6 elicits a chain of events whereby receptor-bound glycoprotein 130 activates Janus kinase 1 which, in turn, phosphorylates signal transducer and activator of transcription 3 (STAT3). This leads to oligomerization of STAT3, nuclear import, binding to STAT response elements in DNA and regulation of transcription^8^. Several knock out models point to a key role of the IL-6-STAT3 pathway in APR gene regulation^9–12^.

Although the upstream components of the IL-1β and the TNF pathways are different, they both converge to regulate gene transcription by two main routes. First, IL-1β- or TNF-dependent activation of the MAP kinase pathway results in activation of the CCAAT/enhancer binding protein beta (CEBPB) and activator protein 1 (AP-1). Second, these cytokines potently activate the nuclear factor κB (NF-κB) family of transcription factors (TFs). The nuclear import and activation of NF-κB is achieved by cytokine-dependent phosphorylation and subsequent degradation of the inhibitory protein IκB^7^. As with STAT3, the three IL-1β- and TNF-activated TFs were shown to play central roles in the hepatic APR^9, 13–15^.

The multi-layered crosstalk between STAT3 and NF-κB has been extensively studied both generally and in the context of liver^2, 16, 17^. Some studies have suggested a direct interaction between the two TFs but the outcome of this interaction can lead to either gene induction^18^ or repression^19^. In addition, STAT3 was shown to retain NF-κB in the nuclei of cancer cells^20^. However, a recent report challenges these notions by showing that the nuclear localization of the two TFs is unaffected by each other’s activity^21^. Thus, the suggested mechanisms for the STAT3-NF-κB crosstalk are often contradictory and a consensus has not been reached.

TFs regulate gene expression by binding to enhancer elements in DNA. Much of the enhancer landscape is determined during development in a cell type-specific manner. In addition, to respond to a constantly changing environment, many enhancers increase or decrease in activity in response to various stimuli^22–25^. The increase in enhancer activity in differentiated cells is initiated by signal-activated TFs leading to the recruitment of chromatin remodeling complexes, histone modifying enzymes and looping factors. These events eventually result in recruitment of RNA polymerase II (RNAP II) to gene promoters and increased transcription. This mechanism of a dynamically changing enhancer landscape ensures rapid response to environmental stimuli^26^.

In addition to fluctuating enhancer activity, which is dictated by TF binding in response to signals, the manner in which TFs bind to enhancers is also dynamic. In contrast to the long-held view, an accumulating body of work suggests that TFs do not bind DNA for periods of time longer than a few seconds^26, 27^. These observations led to alternative models of TF function^26^. Because TFs constantly exchange with the DNA template, one TF can augment the binding of a second TF indirectly. Indeed, it was shown that even TFs that bind an identical DNA motif do not compete for binding, but rather increase each other’s binding capacity^28^. This effect, termed ‘dynamic assisted loading’, does not require a physical interaction between TFs, but is thought to rely on one TF activating the enhancer by recruiting chromatin remodeling and histone modifying enzymes to the enhancer, thereby making it more accessible to other TFs^29^. The assisted loading mode of action has been proposed to function in a variety of transcriptional programs^30–37^. Together, these studies point to a paramount role of chromatin regulation in the assisted loading model. Assisted loading events are coupled with increases in enhancer activity as measured by DNase hypersensitivity or H3K27 acetylation (H3K27ac), two well-established markers for active enhancers^38–40^. Thus, enhancer activation is a central part in TF cooperation via assisted loading. In cases where overt gene induction does not accompany enhancer activation, the enhancer is considered ‘primed’ for a secondary signal.

Due to their critical role in innate immunity and systemic inflammation, transcriptional regulation imposed by pro-inflammatory cytokines in liver is extensively studied^2, 15^. While most studies focused on an individual pathway, some also dealt with probable crosstalk between the major pathways activated in liver during acute or chronic inflammation. Several models have been suggested^2, 16^ but the events bringing about the hepatic immune response during inflammation remain elusive.

Here we report a genome-wide effort integrating transcriptomics, enhancer mapping and TF occupancy profiles aimed at deciphering cytokine crosstalk during inflammation. We show that following a pro-inflammatory signal, TFs cooperate on a specific subset of enhancers via assisted loading to induce a synergistic gene expression program in hepatocytes. Conversely, these TFs do not cooperate, and even antagonize each other’s activity, in other enhancers and target genes. This enhancer-specific crosstalk between TFs reconciles the seemingly contradictory observations of pro-inflammatory TFs in liver^2^ and reveals the mode of action behind inflammatory gene regulation.

## Results

### Combinatorial cytokine stimuli lead to a bifurcated response

**Fig. 1:**
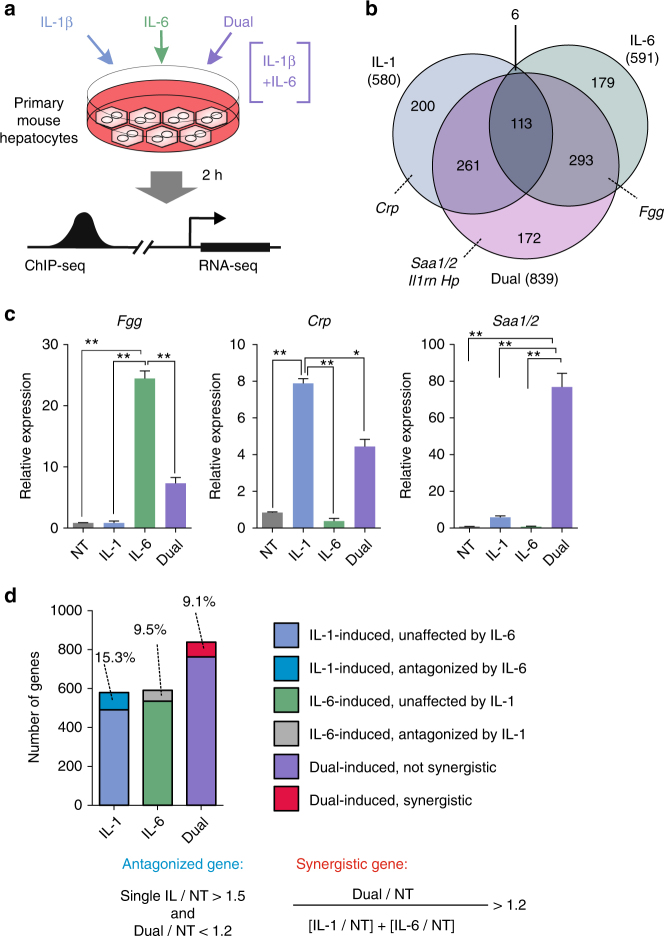
A multifaceted response of primary hepatocytes to pro-inflammatory cytokines IL-1β and IL-6. **a** Experimental setup used throughout the study. Primary mouse hepatocytes were treated with IL-1β, IL-6 or both for 2 h (cytokine conc. 10 ng ml^−1^). Then, cells were collected for downstream experiments. **b** Venn diagram portraying cytokine-induced genes and their different response under the various treatments. In parentheses is the total number of genes induced under each condition (1.5-fold change, adjusted *p*-value ≤0.05, measured with CuffDiff). Prominent APR genes are highlighted. For more details and for repressed genes, see Supplementary Data 1. **c** RNA levels of cytokine-induced genes in primary hepatocytes following a 2 h cytokine treatment. Both antagonistic and synergistic relationships between IL-1β and IL-6 are shown. To more reliably measure changes stemming from transcriptional regulation and not RNA stability, nascent transcripts, rather than mature ones, were measured by quantitative PCR (qPCR) throughout the study. To achieve this, the amplified gene region spans exon-intron junctions in all examined genes. All qPCR gene measurements in the study are relative (the NT sample in each experiment was set with the value 1) and were normalized with the housekeeping gene *Tbp*. Representative experiment shown of at least three independent repeats. Error bars represent s.d. of three technical replicates. Asterisks denote statistical significance as determined by an unpaired, two-tailed t-test. Single asterisk denotes *p* value ≤ 0.05, double asterisks denote *p*-value ≤0.01. **d** The fraction of antagonistically-regulated and synergistically induced genes in each treatment group is presented. Cutoff criteria for antagonism and synergism are shown

During inflammation, hepatocytes are exposed to IL-1β, TNF and IL-6. We employed RNA-seq to evaluate the effects of pro-inflammatory cytokines on hepatic gene expression. To separate individual from combinatorial effects we treated primary mouse hepatocytes with a single cytokine (IL-1β or IL-6) or a combination of the two for 2 h. (Fig. 1a). The same protocol was performed for TNF, a cytokine that activates TFs similarly to IL-1β (Supplementary Fig. 1a). The hepatic transcriptome was dramatically altered following these treatments, with differential expression of 3,260 genes in response to at least one treatment (1.5-fold change, adjusted *p-*value ≤ 0.05, measured by CuffDiff, Supplementary Data 1, Supplementary Fig. 1b).

To validate that our experimental approach recapitulates the changes in hepatic transcriptome seen in *bona-fide* acute inflammatory conditions, we compared the genes induced in the dual treatment (IL-1β + IL-6) to the hepatic transcriptome of mice where both cytokines are elevated following an inflammatory signal. We found a significant enrichment of dual-induced genes within genes induced in mice following either lipopolysaccharide (LPS) injection or *Streptococcus pneumoniae* infection, (Supplementary Fig. 1c). To further relate our findings to chronic inflammation, we employed two datasets where the human liver transcriptome was measured during non-alcoholic steatohepatitis (NASH) or viral hepatitis C (HCV) infection. As found in acute inflammation, dual-induced genes were significantly enriched within NASH- and HCV-induced genes (Supplementary Fig. 1c). We conclude that genes induced following dual IL-1β + IL-6 treatment in primary mouse hepatocytes faithfully represent gene expression patterns seen in acute and chronic inflammation. This is consistent with these inflammatory states showing increased IL-1β and IL-6 levels^1, 9, 41, 42^.

The gene induction patterns of the individual treatments varied considerably between the two cytokines, as expected from the different pathways activated by IL-1β and IL-6. Notably, the induction of many genes induced in the individual treatments was negated in the dual treatment. Reciprocally, a significant portion of dual-induced genes was not induced in individual treatments (Fig. 1b, Supplementary Data 2). To quantify this inter-cytokine crosstalk, we performed k-means clustering of all differentially expressed genes (i.e. all genes with a ≥ 1.5-fold change in expression following at least one treatment, adjusted *p*-value ≤ 0.05, measured by CuffDiff, n = 3,260). This revealed an intricate crosstalk between pro-inflammatory cytokines. With regards to gene induction, three patterns were observed when comparing the dual to single treatments: two ‘antagonistic’ patterns wherein IL-1β and TNF reduced the expression of IL-6-induced genes, and reciprocally, one profile wherein IL-6 reduces the expression of IL-1β- and TNF-induced genes. In addition, a synergistic pattern in which the cytokines enhance each other’s gene induction capacity was also prominent (Fig. 1c, Supplementary Fig. 2a, b, Supplementary Data 1). The effect of TNF on gene expression was similar but much weaker than that of IL-1β (Supplementary Fig. 2a, c). Therefore, the rest of our study focused on crosstalk between IL-1β and IL-6.

While gene clustering can efficiently suggest patterns of gene expression, genes grouped in one cluster do not necessarily pass strict statistical criteria for the observed pattern of the cluster. To more robustly characterize the crosstalk between the two cytokines, we defined antagonistic and synergistic gene groups according to clear criteria. An IL-1β-induced gene (fold change over NT≥1.5) was determined to be antagonized by IL-6 if the fold change in the dual treatment over NT is ≤1.2. The same criteria were applied to IL-6-induced genes. Genes were considered synergistically induced if their fold change in the dual condition is ≥1.2 over the additive fold change of IL-1β and IL-6. These threshold criteria reduced the list of antagonistic and synergistic genes found in the gene clustering to a more clearly-defined set of genes (Fig. 1d, Supplementary Data 2). K-means clustering of only genes that met these criteria (*n* = 221) showed a more robust pattern of synergy and antagonism (Supplementary Fig. 2d).

To gain insight as to the functional roles dual-induced and synergistic genes play in liver biology we analyzed the two gene lists with Ingenuity Pathway Analysis. Acute phase signaling was the most enriched pathway in dual-induced genes (*p*-value = 1.11 × 10^−24^_,_ Fisher’s exact test) as well as in the sub-group of synergistic genes (*p*-value = 2.15 × 10^−10^ Fisher’s exact test). These observations are consistent with the prominent role of the liver in the APR. Thus, most of the individual genes and gene loci analyzed in this study are central *APR* genes. Collectively, these data point to a multifaceted crosstalk between pro-inflammatory cytokines, leading to several gene regulation patterns playing a role in the hepatic inflammatory response.

### Cytokines promote global changes in enhancer activity

**Fig. 2:**
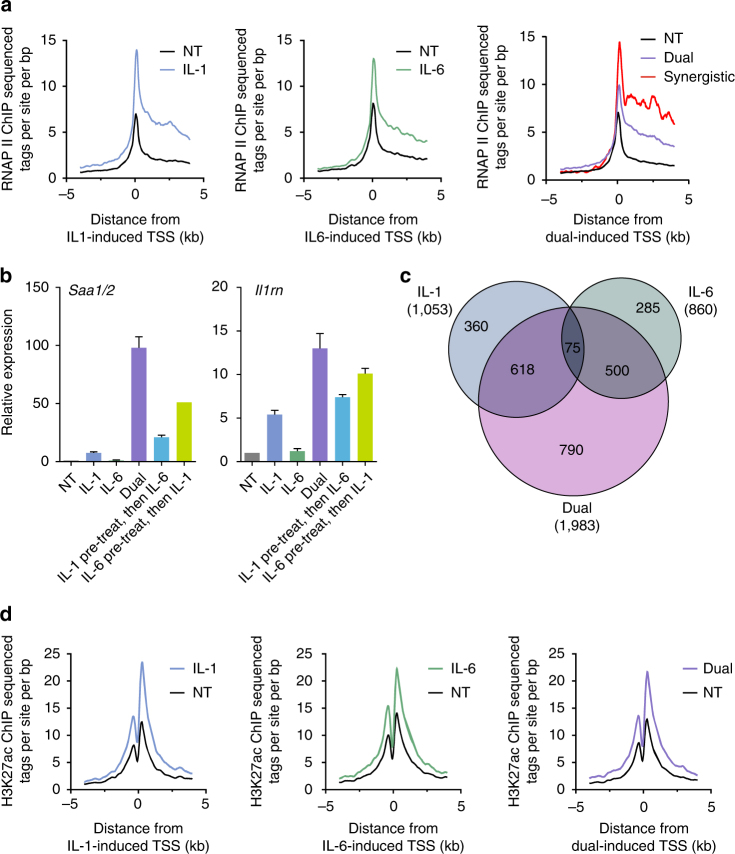
Cytokines globally alter hepatocyte enhancer dynamics and RNAP II occupancy. **a** RNAP II occupancy in cytokine-induced genes increases in a cytokine-dependent manner. **b** Nascent RNA levels of *Saa1/2* and *Il1rn* in primary hepatocytes sequentially treated with either IL-1β or IL-6 for 6 h followed by a 2 h treatment with the second cytokine. Compare to simultaneous 2 h treatment (dual). Representative experiment shown of at least three independent repeats. Error bars represent s.d. of three technical replicates. **c** Venn diagram portraying cytokine-induced enhancers and their different response under the various treatments. Enhancers were defined as genomic regions enriched with H3K27ac in accordance with common practices in the field. Throughout the study, analyses include all H3K27ac regions (both regions proximal and distal to transcription start sites). **d** H3K27 acetylation in the vicinity of cytokine-induced genes increases in a cytokine-dependent manner. TSS transcription start site

Changes in gene expression patterns are caused either by altering gene transcription or by post-transcriptional events such as changes in RNA stability, translational regulation, etc. To find if the changes in gene expression we observed are due to transcriptional regulation, we assayed RNAP II occupancy following cytokine treatment by chromatin immunoprecipitation sequencing (ChIP-seq). RNAP II occupancy at the transcriptional start site and in the gene body of IL-1β-induced genes was increased following IL-1β treatment. The same correlation was found in IL-6- and dual-induced genes, suggesting that cytokine-dependent gene induction is mediated primarily by changes in gene transcription. Interestingly, synergistic genes showed more RNAP II occupancy compared to the average occupancy of all dual-induced genes combined and compared to single treatments (Fig. 2a, Supplementary Fig. 2e).

The complex crosstalk between IL-1β and IL-6 leading to both antagonistic and synergistic gene expression implies that several separate mechanisms downstream of these cytokines are at play during inflammation, each dictating a different gene expression pattern. Of particular interest was the synergistic group of genes due to their prominent role in the APR. Two major scenarios might bring about a synergistic pattern of gene expression. In the first scenario, a TF cascade is initiated when one cytokine induces the transcription of the second cytokine’s activated TF. Alternatively, a direct scenario is plausible in which the two cytokines independently activate TFs which then cooperate with each other, resulting in a synergistic effect. To differentiate between these two options, we pre-treated cells with either cytokine followed by a subsequent treatment with the second cytokine. In the case of a TF cascade, the pre-treated samples would have increased levels of the secondary TF and thus show increased synergism compared to concomitantly-treated samples. However, in all six examined genes, the pre-treatment did not enhance the response, and in some cases even reduced it (Fig. 2b, Supplementary Fig. 3a; the expression of these six synergistic genes are examined throughout the study). Moreover, simultaneous dual treatment for prolonged periods of time did not change expression patterns which peaked at 2 h and waned thereafter (Supplementary Fig. 3b). These observations suggest cytokine-activated TFs directly cooperate, possibly on the chromatin template, to induce genes.

Given these findings, we turned our attention to the active enhancer repertoire of hepatocytes and how it changes following cytokine treatment. To map enhancers, we mapped H3K27ac genome-wide by ChIP-seq. In agreement with previous studies and with its tight association with gene regulation, H3K27ac peaks were most abundant in intergenic and intronic regions (Supplementary Fig. 4a; in this analysis and all subsequent analyses, all H3K27ac regions were included—both regions proximal and distal to transcription start sites). Moreover, the H3K27ac ChIP-seq signal in primary hepatocytes was associated with enhancer marks in mouse liver (DNase hypersensitivity and p300 occupancy), suggesting that hepatocytes largely recapitulate enhancer regions of intact mouse liver (Supplementary Fig. 4b). When profiling changes in H3K27ac between different cytokine treatments, we found that many enhancers were activated following the various treatments in a pattern reminiscent of gene induction patterns (Fig. 2c). Indeed, the H3K27ac signal increased in the vicinity of cytokine-induced genes in a cytokine-specific manner (Fig. 2d). Further attesting to the cytokine-dependent activation of these enhancers, RNAP II occupancy at these enhancers increased in a cytokine-specific manner as well (Supplementary Fig. 4c). This finding is in line with reports establishing RNAP II occupancy as a marker for enhancer activity^43, 44^. Taken together, these data show that pro-inflammatory cytokines dramatically alter the hepatic chromatin landscape to facilitate global changes in gene transcription.

To gain insight as to the major TFs occupying these enhancer groups, we performed motif enrichment analysis. IL-1β-induced enhancers were enriched for motifs bound by the CEBP, AP-1 and NF-κB TFs, while IL-6-induced enhancers were enriched for STAT3 and ATF3 motifs. The top four motifs enriched in dual-induced enhancers are bound by CEBP, AP-1, NF-κB and STAT3 (Supplementary Fig. 4d). Except for ATF3, which antagonizes the IL-6 pathway^45, 46^, the enriched motifs precisely reflect the TFs known to be activated by IL-1β (CEBP, AP-1 and NF-κB) and IL-6 (STAT3) and promote inflammation^2, 14, 15^. Interestingly, IL-6 induces the gene levels of STAT3 (3.3-fold), cJun (the major subunit of AP-1, 1.8-fold) and CEBPB (1.9-fold) while IL-1β induces the gene level of p65 (the principal hepatic NF-κB subunit^7^, 1.7-fold, Supplementary Data 2). While these gene inductions may have effects during chronic inflammation, they do not affect gene regulation in the first 24 h of cytokine exposure (Fig. 2b, Supplementary Fig. 3a, b).

### STAT3 binding is augmented by IL-1β in a subset of enhancers

**Fig. 3:**
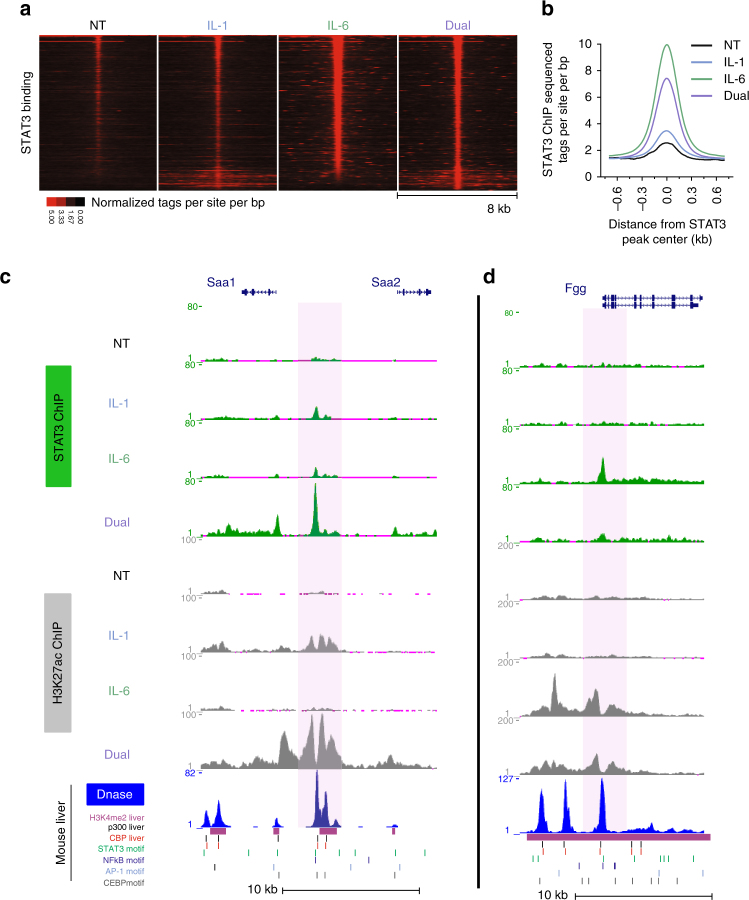
STAT3 binding is generally increased by IL-6 but is augmented by IL-1β at a subset of enhancers. **a** Heat map depicting STAT3 binding intensity at all STAT3 sites (sorted from high to low in the IL-6 condition). From a total of 2,975 peaks, 1.2% peaks (*n* = 39) were called in non-treated cells, 8.3% (*n* = 247) in IL-1-treated cells, 39.7% peaks (*n* = 1182) in IL-6-treated cells and 59.5% peaks (*n* = 1772) in dual-treated cells (see also Supplemental Data 3). **b** Overall STAT3 binding increases following IL-6 treatment. **c**, **d** Genome browser tracks showing IL-1β-dependent activation of the *Saa1/2* enhancer and the dependence in IL-1β for maximal STAT3 binding (**c**). In contrast, at the *Fgg* locus, STAT3 binding is maximal in the presence of only IL-6 and is reduced upon IL-1β addition (**d**). H3K27ac sites overlap with key enhancer marks: DNase hypersensitive sites, p300/CBP binding sites and H3K4me2. DNase hypersensitivity data adopted from Goldstein et al.^37^ p300/CBP data adopted from Faure et al.^68^ and H3Kme2 data adopted from Sun et al.^67^

**Fig. 4:**
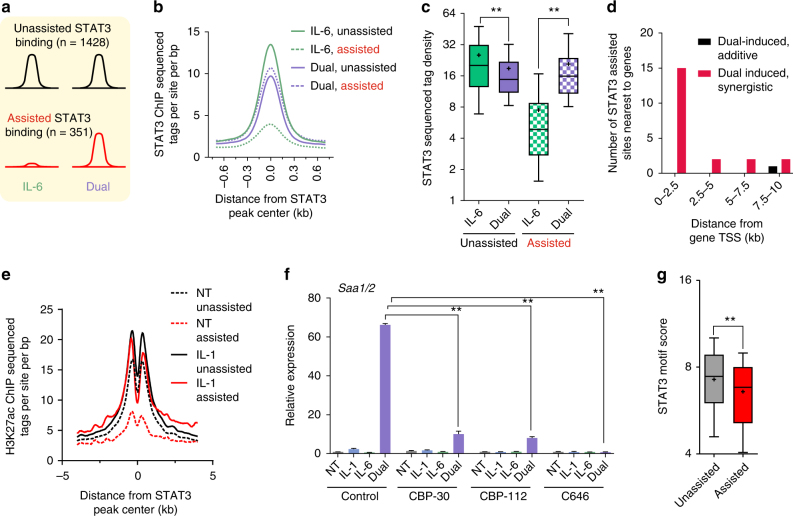
IL-1β primes a subset of enhancers and assists STAT3 loading onto them. **a** A scheme representing the fraction of STAT3 sites where no significant change in binding was detected between the IL-6 and dual conditions (‘unassisted sites’) and the fraction of sites where a significant (fold change ≥1.7, *p*-value ≤0.01, measured with DESeq) increase in binding was found in dual-treated cells compared to IL-6-treated cells (‘assisted sites’). **b**, **c** Assisted STAT3 binding sites only reach maximal binding in the dual treatment whereas STAT3 binding at unassisted sites is higher in IL-6-treated cells. **d** The number of assisted STAT3 binding sites nearest to either synergistic genes or to the same number of dual-induced genes with an additive fold change. Plotted as a function of distance from gene transcriptional start site (TSS, plotted in 2.5 kb bins). Only the nearest site is counted, second- or third-nearest sites are not shown. **e** H3K27ac ChIP-seq tag density in the vicinity of STAT3 binding sites in the non-treated vs IL-1β conditions showing that IL-1β activates enhancers where assisted sites reside. **f** Nascent RNA levels of *Saa1/2* in primary hepatocytes pre-treated with p300/CBP inhibitors followed by indicated cytokine treatments. Representative experiment shown of at least three independent repeats. Error bars represent s.d. of three technical replicates. **g** Box plot depicting the STAT3 motif score in assisted vs unassisted sites. Double asterisks denote *p*-value ≤0.01 as determined by an unpaired, two-tailed *t*-test

As our motif analysis suggested, IL-6 regulates transcription mainly by STAT3, while the transcriptional response regulated by IL-1β was divided to three different TFs. This, together with the well-established role of STAT3 in hepatic inflammatory processes, and the concept that it is the primary APR TF^2, 8, 47^, made it the first candidate for further investigation. Accordingly, an inhibitor of STAT3 activity dramatically perturbed synergistically induced genes (Supplementary Fig. 5a). To examine the global effect of STAT3 during inflammation, we profiled its genome-wide occupancy following cytokine treatment using ChIP-seq. As expected, STAT3 binding was markedly increased following IL-6 treatment (Figs. 3a, b Supplementary Data 3). Surprisingly, at a subset of sites, STAT3 binding was substantially increased in the dual treatment compared to IL-6 (Fig. 3c, Supplementary Fig. 5b). Conversely, STAT3 binding at some sites was perturbed in the dual treatment (Fig. 3d). When quantifying this phenomenon, we found 351 sites (20% of total STAT3 binding events) where STAT3 binding was significantly increased in the dual treatment compared to IL-6. In contrast, the majority of sites (80%, *n* = 1428) were unaffected, or even reduced by addition of IL-1β (Fig. 4a, Supplementary Data 4). Because IL-1β does not directly activate STAT3 but is still essential for maximal STAT3 binding at a subset of sites, we termed these ‘assisted sites,’ while the binding sites not requiring IL-1β were termed ‘unassisted sites’ (Fig. 4a). When examining the extent of STAT3 binding, we found that IL-1β does not lead to ‘super’ STAT3 binding at assisted sites. Rather, IL-1β allows STAT3 binding at assisted sites to reach the level of unassisted sites (Figs. 4b, c). In stark contrast to assisted sites, addition of IL-1β led to a decrease in STAT3 binding at unassisted sites (Figs. 4b, c). Taken together, these findings reveal that while IL-6 is sufficient to efficiently activate STAT3 at most sites, IL-1β is required for maximal STAT3 binding in 20% of sites, while reducing it at others.

### Assisted STAT3 sites are primed by IL-1β

The pattern of STAT3 binding at assisted sites, where maximal binding is reached only with the dual treatment, is reminiscent of the pattern observed in the synergistic gene induction group. To learn whether the two phenomena are correlated, we examined the proximity of assisted sites to synergistic genes. Assisted STAT3 peaks were far more likely to reside proximally to synergistic genes than to the same number of dual-induced genes with an additive fold change (Fig. 4d). Reciprocally, the gene nearest to 63 assisted sites was a synergistic gene compared to only 2 additive genes (Supplementary Data 5). Often, these sites resided kilobases away from the transcription start site, a common phenomenon in genome-wide TF occupancy profiles (Supplementary Fig. 5c).

These findings suggest a specific mode of action dedicated for synergistic gene induction whereby an IL-1β-activated TF assists STAT3 loading on specific enhancers associated with synergistic genes. Support for that model came from the analysis of H3K27ac marks at assisted sites. We reasoned that because IL-1β assists STAT3 binding only on a subset of sites, the augmenting effect of IL-1β is exerted at those enhancers and not upstream by a global activation of STAT3 (which would result in augmented activity in all binding sites). Thus, we examined the enhancer state of STAT3 binding sites following IL-1β treatment. The H3K27ac signal at unassisted sites was similar between the non-treated and IL-1β-treated conditions. However, the H3K27ac signal at assisted sites only reached a level similar to unassisted sites following IL-1β treatment (Figs. 3c, 4e, Supplementary Fig. 5b). Thus, enhancers harboring assisted sites are somewhat quiescent in the untreated condition and are ‘primed’ for STAT3 binding by IL-1β. Because robust H3K27ac facilitates gene expression^48^, we examined the effect of enhancer priming on synergistic gene expression. To that end, we inhibited the two major enzymes catalyzing H3K27 acetylation, p300 and CREB binding protein (CBP). Both genome-wide studies and functional observations directly link p300/CBP occupancy with increased H3K27ac^49–52^. Synergistic gene expression was either completely abolished or severely diminished following two of the p300/CBP inhibitors used (SGC-CBP30 and C646). The third inhibitor (I-CBP112) diminished synergy in four out of six tested genes (Fig. 4f, Supplementary Fig. 6).

These data imply that the enhancers where assisted sites reside are less active prior to IL-1β treatment, a condition unfavorable to STAT3 binding. Additionally, the ability of STAT3 to bind its motif is probably further diminished due to the low average motif score in assisted sites compared to unassisted sites (Fig. 4g). Collectively, these findings support a scenario whereby STAT3 binding at assisted sites is perturbed in the lack of IL-1β due to diminished enhancer activity but is increased following enhancer activation by an IL-1β-activated TF, resulting in synergistic gene expression.

### IL-1β-activated NF-κB preferentially binds assisted sites

**Fig. 5:**
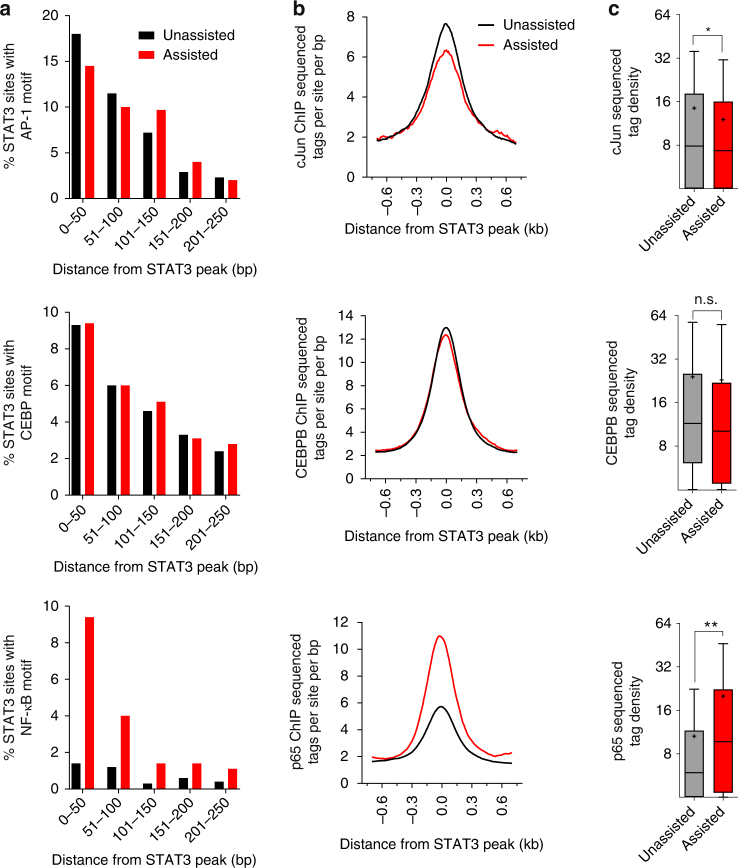
p65 but not CEBPB or cJun preferentially binds at assisted sites. **a** The percent of STAT3 binding sites flanking AP-1, CEBP and NF-κB motifs shows an enrichment of NF-κB motifs in the vicinity of assisted sites. Plotted as a function of distance of motif from center of STAT3 binding site (in 50 bp bins). **b**, **c** cJun, CEBPB and p65 binding following IL-1β treatment in the vicinity of assisted or unassisted STAT3 binding sites shows an enrichment of p65 binding next to assisted sites. Asterisks denote statistical significance as determined by an unpaired, two-tailed *t*-test. Single asterisk denotes *p*-value ≤0.05, double asterisks denote *p*-value ≤0.01. NS denotes not significant

After establishing a priming effect for IL-1β in assisting STAT3 binding, we set out to isolate the IL-1β-activated TF responsible for it. As suggested by the motif enrichment analysis (Supplementary Fig. 4d) and by previous reports^9, 13–15^ the major TFs activated by IL-1β are AP-1, CEBPB and NF-κB. Therefore, we examined the motif occurrence of these TFs next to STAT3 binding sites. While the CEBP and AP-1 motifs occurred at a similar frequency in assisted and unassisted sites, the NF-κB motif was much more prevalent in assisted sites (Fig. 5a). Then, we profiled the genome-wide binding of these TFs following IL-1β treatment using ChIP-seq. In line with motif occurrence, binding of cJun and CEBPB was similar throughout STAT3 binding sites (cJun binding was modestly increased in unassisted sites). Conversely, the binding of the NF-κB subunit p65, was significantly enriched around assisted STAT3 sites compared to unassisted sites (Fig. 5b, c).

### NF-κB primes assisted sites and facilitates STAT3 binding

**Fig. 6:**
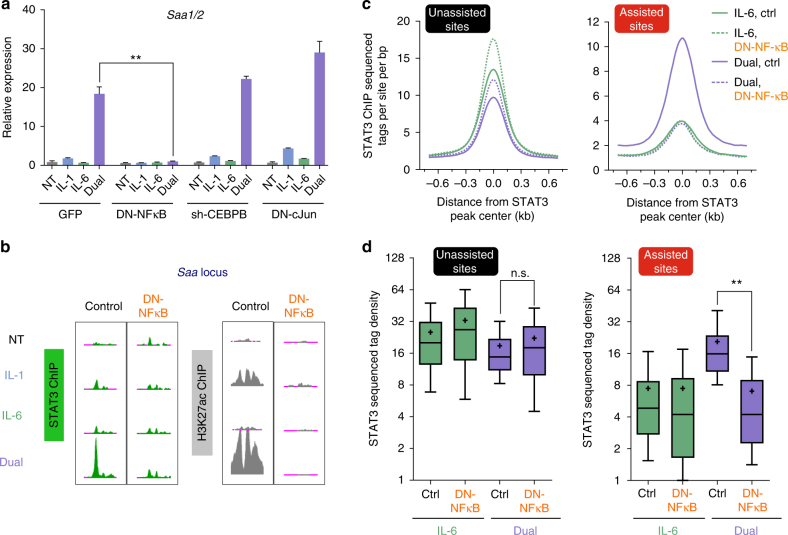
NF-κB is responsible for IL-1β-mediated assisted STAT3 binding and synergistic gene expression. **a** Nascent RNA levels of *Saa1/2* in primary hepatocytes pre-treated with adenovirally-expressed inhibitors of IL-1β-activated TFs. Representative experiment shown of at least three independent repeats. Error bars represent s.d. of three technical replicates. **b** Genome browser tracks depicting STAT3 assisted loading as well as enhancer priming at the *Saa1/2* locus are negated in the presence of a dominant negative peptide inhibiting NF-κB activity (DN-NFκB). **c**, **d** The IL-1β-dependent increase in STAT3 binding at assisted sites is negated in the presence of a dominant negative peptide inhibiting NF-κB activity. Asterisks denote statistical significance as determined by an unpaired, two-tailed t-test. Single asterisk denotes *p*-value ≤0.05, double asterisks denote *p*-value ≤0.01. NS denotes not significant

These findings correlate NF-κB, but not CEBPB or AP-1, to assisted loading of STAT3. To explore this causally, we inhibited the activity of the three TFs via adenoviral vectors expressing either dominant negative peptides (targeting AP-1 and NF-κB) or small hairpin RNA (targeting CEBPB). Synergistic gene induction was unaffected by perturbed CEBPB or AP-1, but was abolished following NF-κB inhibition (Fig. 6a, Supplementary Fig. 7a). In contrast, the expression of *Fgg*, an IL-6-induced gene antagonized by IL-1β, was increased in NF-κB-inhibited cells (Supplementary Fig. 7b). Next, we profiled STAT3 binding in the presence of a NF-κB dominant negative peptide (DN-NFκB). Remarkably, assisted loading of STAT3 was completely negated in most regions under those conditions (Fig. 6b–d, Supplementary Fig. 8a). In fact, 92% of sites (325/351) did not show any STAT3 binding when NF-κB was inhibited (Supplementary Data 4). In stark contrast, the level of STAT3 binding at unassisted sites was unaffected (and even sometimes increased) by DN-NFκB, suggesting a highly enhancer-specific augmenting effect of STAT3 binding by NF-κB (Fig. 6c, d, Supplementary Fig. 8b, c). This enhancer-specific, rather than systemic effect is supported by the finding that IL-1β treatment did not lead to increases in the total gene or protein levels of STAT3 and did not increase the levels of STAT3 phosphorylation on Tyr705, a modification correlated with activity (Supplementary Fig. 8d, Supplementary Data 2).

**Fig. 7:**
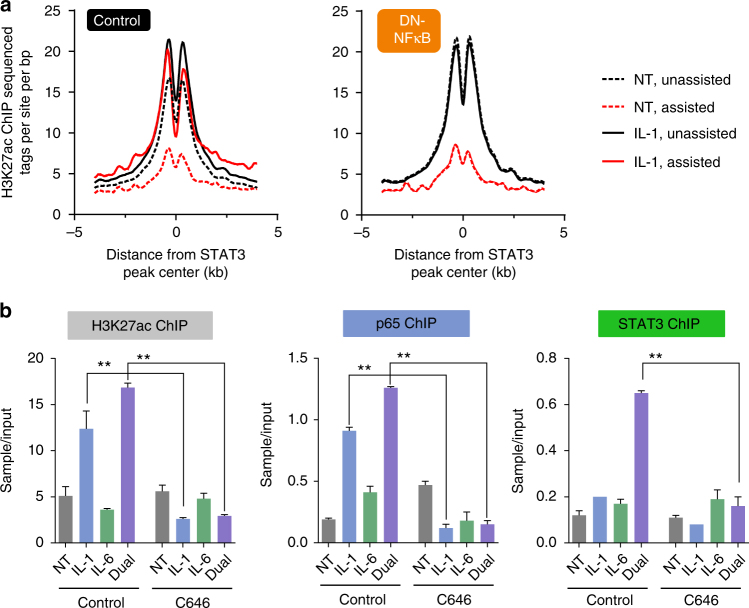
IL-1β enhancer priming is mediated by NF-κB and is brought about by p300/CBP-dependent H3K27ac. **a** The IL-1β-dependent increase in H3K27 acetylation at assisted sites is negated in the presence of a dominant negative peptide inhibiting NF-κB activity (left panel replicated from Fig. 4e). **b** ChIP-qPCR of the *Saa1/2* enhancer shows that assisted loading as well as H3K27ac is negated in the presence of a p300/CBP inhibitor (C646). Representative experiment shown of two independent repeats. Error bars represent s.d. of three technical replicates. Double asterisks denote *p*-value ≤0.01 as determined by an unpaired, two-tailed *t*-test

Because assisted enhancers are primed with H3K27ac following IL-1β, we examined if NF-κB plays a role in that aspect as well. Indeed, in the presence of dominant negative NF-κB, the priming effect IL-1β has on assisted enhancers was abolished, keeping H3K27ac at a level similar to that of non-treated cells (Fig. 7a). As shown above, the enzymes catalyzing H3K27ac (p300/CBP) are involved in synergistic gene expression (Fig. 4f, Supplementary Fig. 6). Moreover, NF-κB is able both to recruit p300/CBP to chromatin^53^ and to increase H3K27ac^54, 55^. Thus, we reasoned that inhibiting these enzymes would perturb both H3K27 acetylation and assisted STAT3 binding. Indeed, in control cells, STAT3 binding was assisted by IL-1β and associated with a marked increase in H3K27ac. However, when p300/CBP were inhibited, both H3K27 acetylation and STAT3 binding were negated (Fig. 7b). Collectively, these findings implicate NF-κB and H3K27ac in STAT3 assisted loading at a subset of enhancers.

## Discussion

The liver plays a far-reaching role in innate immunity and the response to pathogens, tissue injury and various trauma. Early during an inflammatory response, hepatocytes are exposed to pro-inflammatory cytokines (chief among them are IL-1β and IL-6). A cascade of intracellular events leads to secretion of a myriad of plasma proteins with various roles in suppressing infection and alleviating trauma and tissue damage^1, 2^. Assaying the individual vs combinatorial effect of these cytokines in a homogeneous and isolated cell system and in a genome-wide manner led us to several discoveries. The combinatorial response of inflammatory cytokines is bifurcated, with some genes synergistically induced while others are antagonized compared to the single cytokine treatments. Our time course and pre-treatment experiments suggest that these effects are mediated by crosstalk between TFs rather than a TF cascade or other mechanisms of temporal organization. Instead, our study reveals an enhancer-specific type of cooperation between cytokine-activated TFs. We found that assisted STAT3 sites have distinct determinants that distinguish them from unassisted sites and provide this enhancer-specific mode of action: (a) STAT3 binding is very low following IL-6 treatment and only reaches maximal binding in the dual treatment (in contrast to unassisted sites that follow the prevalent dogma, i.e. that IL-6 treatment is sufficient for maximal STAT3 binding). (b) H3K27ac is low at the untreated condition and increases markedly following IL-1β treatment. (c) The STAT3 motif is weaker in these enhancers compared to unassisted enhancers. (d) These enhancers are proximal to synergistic genes. (e) NF-κB motifs and p65 binding are enriched in these enhancers compared to unassisted enhancers. (g) Synergistic gene expression, H3K27ac priming and STAT3 binding are negated in these enhancers following inhibition of NF-κB.

**Fig. 8:**
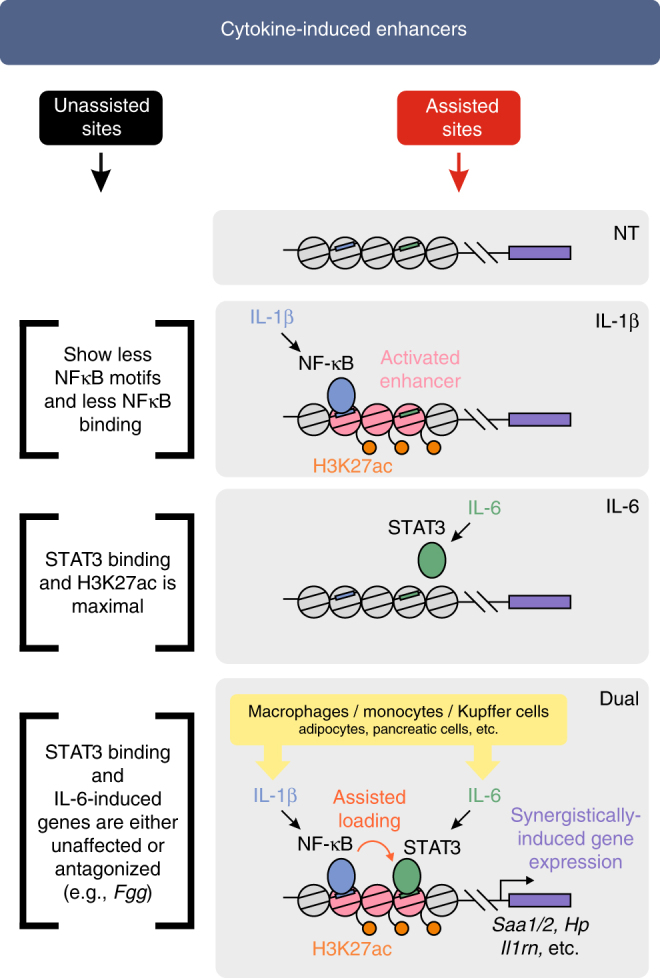
Enhancer priming and assisted loading of STAT3 by NF-κB promotes synergistic gene expression. Upon IL-1β treatment, NF-κB binds a subset of ‘assisted enhancers’ (right-hand side), leading to increased p300/CBP-mediated H3K27ac. This event does not lead to prominent gene induction, rather, it primes the enhancer for STAT3 binding which would only be weakly bound without priming (in the IL-6 only treatment). When hepatocytes are exposed to both IL-1β and IL-6 (such as in acute and chronic inflammation), NF-κB assists STAT3 binding, leading to synergistic gene expression. Conversely, the enhancers where most STAT3 sites reside are not primed by NF-κB (‘unassisted sites’, left-hand side) and STAT3 achieves maximal binding in the presence of only IL-6

Collectively, these observations support a model whereby upon IL-1β stimulation, NF-κB is activated and binds enhancers near synergistic genes. This leads to a switch in the enhancer state from a quiescent to an activated one. Due to this enhancer priming, STAT3 binds these enhancers much more efficiently in the dual treatment than it would in the presence of only IL-6. These events lead to synergistic expression of APR genes. In contrast, at unassisted sites, NF-κB does not enhance (and in some cases even perturbs) STAT3 binding (Fig. 8).

The crosstalk between STAT3 and NF-κB is an active field of research with studies showing contradictory outcomes of concomitant activation of the two TFs. Some suggest cooperation between them, while others show antagonism^2, 16–21^. Our results reconcile some of the confusion in the field by showing that a significant portion of STAT3-NF-κB cooperation occurs in an enhancer-specific manner rather than in a systemic one. Indeed, neither STAT3 gene and protein expression, nor total chromatin-binding levels are affected by IL-1β in our system. Conversely, STAT3 binding is dramatically affected at a subset of enhancers near synergistic genes. A model based solely on protein–protein interaction cannot explain enhancer-specificity whereby NF-κB promotes STAT3 binding at some enhancers, while perturbing it at others. We suggest that assisted loading of STAT3 by NF-κB is at play during inflammation, allowing a bifurcated gene regulation program and a fine-tuned crosstalk between the two TFs. Assisted loading has been described in various systems^30–37^. This report expands the reach of that model to immunological responses. Our study also sheds light on previously described immune-related transcriptional programs where assisted loading was not suggested but seems plausible in light of our findings^24, 56, 57^.

The dynamic and signal-dependent nature of enhancer activation seen in this study, and in many others, is at odds with the concept that the enhancer landscape is set during differentiation and remains rigidly static thereafter. The ability of certain TFs to establish tissue-specific enhancer landscapes is not under dispute. However, these TFs (termed pioneer factors) were attributed with special properties that separated them functionally from the rest of TFs^58^. Mainly, they are claimed to open inaccessible chromatin without the assistance of chromatin remodelers. This has been recently challenged by us and others^59^. For example, the chromatin loading of FoxA1 (a well-established pioneer factor), was shown to need assistance by non-pioneer TFs in a signal-dependent manner^35^. Moreover, pioneer factors have been shown to be dependent on chromatin remodelers during differentiation^60^. Thus, it appears that pioneer factors play a role in establishing chromatin landscape in a manner dependent on both chromatin remodelers and assisted loading by signal-activated TFs. These observations are consistent with a reported role for NF-κB (a classic signal-activated TF) in modifying the enhancer landscape following stimuli^54, 55^. These reports, together with ours, challenge the concept that NF-κB and other signal-activated TFs are restricted to enhancer regions pre-activated by lineage-determining factors^58^. Considering this, assisting of NF-κB by STAT3 is also plausible but we found no IL-6 enhancer priming next to synergistic genes, thereby making this option less likely.

In summary, the findings we report here reveal a regulatory module in inflammatory signaling whereby NF-κB primes a subset of enhancers and assists STAT3 loading on them. These two TFs then synergistically induce acute phase genes critical to protect from infection and tissue damage. We propose that such a dynamic assisted loading mechanism is suitable for the demand for rapid and intense gene induction during the APR. Conversely, the rest of STAT3 binding sites are either unaffected or even suppressed by NF-κB. This phenomenon resolves some of the confusion regarding the complex crosstalk between the two TFs and unfolds critical regulatory events in acute and chronic inflammation.

## Methods

### Reagents and treatment regimens

Cytokines (R&D systems) were reconstituted in PBS 0.1% BSA. Treatment durations and final concentrations were optimized and set at 2 h for the lowest concentrations leading to maximal gene induction (IL-1β and IL-6 at 10 ng ml^−1^, TNF at 2 ng ml^−1^). p300/CBP inhibitors C646, SGC-CBP30 and I-CBP112 (Sigma, Cat# SML0002, SML1133 and SML1134 respectively) were reconstituted in DMSO and treated at a final concentration of 35 µM 20 min. prior to cytokine treatment. STAT3 inhibitor (BP-1-102, Sigma, Cat# 573132) was reconstituted with DMSO and treated at a final concentration of 30 µM 20 min. prior to cytokine treatment. Adenoviral vectors targeting AP-1, NF-κB and CEBPB (Vector Biolabs, Cat# 1046, 1028 and shADV-255244, respectively) were added to hepatocytes 4 h after plating (18 h prior to cytokine treatments) at a final concentration of 10^6^ pfu per 35 mm well (for qPCR) or 100^6^ pfu per 15 cm plate (for ChIP)

Antibodies used: STAT3, p65 and CEBPB (Santa Cruz biotechnology, sc-482X, sc-372X, sc-150X, respectively), GAPDH and RNAP II (Abcam, Cat# ab8245 and ab5131, respectively), cJun and phospho-STAT3 Tyr705 (Cell Signaling Technologies, Cat# #9165 and #9145, respectively) and H3K27ac (Active motif #39133).

### Primary mouse hepatocyte isolation and culture conditions

Male C57BL/6 mice (8–12 weeks old, Charles River) were maintained according to NIH guidelines under 12 h light-dark cycles. Hepatocytes isolated by the two-step collagenase perfusion technique^61^. Mice were sacrificed by cervical dislocation and the liver was immediately perfused through the vena cava with HBSS (no calcium, no magnesium, no phenol red, Gibco, 14175-095, 0.5 mM EDTA, 25 mM HEPES, pH = 7.4). Then, the liver was perfused with 30 ml of liver digest medium (Gibco, 17703-034). During perfusion the portal vein was cut and periodically clamped for 10 s. liver was excised into 10 ml of liver digest medium. Liver content was gently released to medium with forceps, spun down at 50 g and spun down again with 1:1 phosphate-buffered Percoll solution (Sigma, P1644) and low glucose DMEM (Gibco 11885-084, 10% FBS) at 200 g, washed with low glucose DMEM, spun at 50 g and plated on BioCoat collagen-coated plates (Corning) with dexamethasone-free plating media (low glucose DMEM+ Gibco, CM3000). 4 h after plating media was changed to dexamethasone-free maintenance media (low glucose DMEM+ Gibco, CM4000).

### RNA isolation and quantitative real-time PCR (qPCR)

Total RNA was isolated from collagen-coated 6-well plates (Corning) using NucleoSpin kit (Macherey-Nagel) according to the manufacturer’s protocol.

For qPCR, a 1 µg aliquot of total RNA was reverse transcribed using iScript cDNA synthesis kit (Bio-Rad, 170-8891). qPCR was performed with a C1000 Touch thermal cycler CFX96 instrument (Bio-Rad) using iQ SYBR Green supermix (Bio-Rad, 170-8887). All qPCR experiments were replicated at least three independent times. Error bars represent s.d. of technical replicates. Gene values were normalized with *Tbp*. The primers used in this study (except for *Tbp*) were designed to amplify nascent transcripts (i.e., the amplified region span exon-intron junctions) as a proxy for transcription to avoid confounding post-transcriptional events. Due to the highly similar sequence of the *Saa1* and *Saa2* genes, the primer used in this study could not differentiate between them (and neither did any other primer we tested).

### Primers used in qPCR

Tbp Fwd: 5′-CCCTATCACTCCTGCCACACCAGC-3′, Rev: 5′-GTGCAATGGTCTTTAGGTCAAGTTTACAGCC-3′

Saa1/2 Fwd: 5′-TCTCAAAGGCATGGGCAGAG-3′, Rev: 5′-TCATGTCAGTGTAGGCTCGC-3′

Il1rn Fwd: 5′-CCTCGGCAATTACCTGACCAT-3′, Rev: 5′-CAGCTGACTCAAAGCTGGTG-3′

Hp Fwd: 5′-AGAGGTCCACGATGAGGTGA-3′, Rev: 5′-GTTCCTGCATCCCAGCTTCT-3′

Fgg Fwd: 5′-AGACTGGAATGGCAGAACCAG-3′, Rev: 5′-ACCAGCTGCAAAGCTCCATT-3′

Crp Fwd: 5′-ATCCCAGCAGCATCCATAGC-3′, Rev: 5′-AAGTTCCGACCATTCTCCCAT-3′

Cxcl9 Fwd: 5′-GTAGTGGATCGTGCCTCGG-3′, Rev: 5′-ACACTCAGTCGCAGCAATAGT-3′

Gbp6 Fwd: 5′-CCAGAGGACCAGTTGGATCAC-3′, Rev: 5′-AGATGTTAACTGGGGCAAGGG-3′

Hamp Fwd: 5′-ATCTCCATCAACAGGTGAGCA-3′, Rev: 5′-TAAGGACCACCCTCTTCCTTGT-3′

### RNA-seq analyses

For RNA-seq, library protocol used was Illumina TruSeq, Epicenter Ribo-Zero (sequencing chemistry Illumina TruSeq 3.0). Significant change of RNA expression between conditions in the RNA-seq experiment was analyzed by Cufflinks and CuffDiff^62, 63^ using UCSC mm9 reference annotation “genes.gtf” with the command cuffdiff -b genome.fa -u genes.gtf. We used “cummeRbund” R package to read and analyze the Cuffdiff results. Two replicates were used for each treatment. Fold change cutoff ≥ 1.5, adjusted p value (q-value) ≤ 0.05, Supplementary Data 2. Replicate reproducibility was high as measured with Pearson correlation (NT: 0.997, IL-6: 0.997, TNF: 0.996, IL-1β: 0.997, IL-6 + TNF: 0.997, IL-6 + IL-1β: 0.994).

For k-means clustering, FPKM data were imported into R for further analyses. All computational analyses were performed on log2 (FPKM + 0.1) values. For cluster analysis, partitions around the medoids (PAM) algorithm was used after gene-specific centering. 3,260 genes whose expression changes by more than 1.5-fold in any stimulation were included for analysis. Clustering was applied to the average of the biological duplicate expression data in each condition. Different numbers of clusters were tested to determine the final number of clusters which represent major patterns in the data. Cluster heatmaps were generated displaying all the RNA-seq duplicate data.

Applying the same parameters, a second k-means clustering was performed using Cluster3.0 and by considering only genes that met threshold criteria, i.e. only synergistic and antagonistic genes (*n* = 221, Fig. 1d, Supplementary Data 2 and their related text).

In the MA plots, The *y* axis represents the difference of log2 FPKM values and the *x* axis stands for the arithmetic mean of log2 FPKM values between two conditions. Points were marked red if the *q*-value of the gene is less than 0.05.

### Ingenuity pathway analysis

Dual-induced genes were analyzed through the use of ingenuity pathways analysis (IPA) (Ingenuity Systems, www.ingenuity.com).

### Gene set enrichment analysis

For gene set enrichment analysis (GSEA)^64^, dual-induced genes were compared to a ranked gene list from four different databases.

### Chromatin immunoprecipitation

Primary hepatocytes (7–10 × 10^6^ cells in a collagen-coated 15 cm plate, Corning) were crosslinked with 1% formaldehyde for 10 min at room temperature and quenched with 0.125 M glycine. Crosslinked cells were washed in PBS, resuspended in chromatin immunoprecipitation (ChIP) lysis buffer (0.5% SDS, 10 mM EDTA, 50 mM Tris-HCl pH8) and sonicated (Bioruptor, Diagenode) to release ~500 bp fragments. Antibodies (4 µg per 100 µg chromatin) were conjugated to magnetic beads (DynaBeads, Invitrogen) for 2 h at 4 °C. Chromatin was pre-cleared for 2 h with unconjugated beads and then immuneprecipitated with beads-Ab conjugates over night at 4 °C. Immunocomplexes were washed sequentially with the following buffers: low-salt buffer (0.01% SDS, 1% Triton x-100, 2 mM EDTA, 20 mM Tris-HCl pH8, 150 mM NaCl), high salt buffer (0.01% SDS, 1% Triton x-100, 2 mM EDTA, 20 mM Tris-HCl pH8, 500 mM NaCl), LiCl buffer (0.25 M LiCl, 1% NP-40, 1% Na-deoxycholate, 1 mM EDTA, 10 mM Tris-HCl pH8) and TE buffer (10mMTris-HCl, 1 mM EDTA pH8). Chromatin was de-proteinized for 2 h at 55 °C (proteinase K, Ambion) and de-crosslinked over night at 65 °C. DNA was subsequently phenol-chloroform purified and ethanol precipitated.

### Primers used for ChIP-PCR

Negative control region (with no TF binding and no H3K27ac):

Fwd: 5′-TGAGCAGGCAGAAATAGGAGC-3′, Rev: 5′-GCTACCATAGTGAGCAAGCCA-3′

Saa 1/2 enhancer (located between Saa 1 and Saa2):

Fwd: 5′-GTGCCCAGTGAGCTCTTCAT-3′, Rev: 5′-CAAGAGACTGCCAAGGCTGA-3′

### Western blot

Cells were lyzed with RIPA buffer and 50 µg of protein was loaded on Mini-PROTEAN TGX stain free gels (4–20% gradient, Bio-Rad), proteins were transferred to PVDF membrane (Trans Blot Turbo, Bio-Rad) and incubated with primary antibody (1:4000 anti-p65, anti-STAT3 and anti-phospho-STAT3, 1:7,500 anti-GAPDH) for 1 h at room temperature. After three washes with Tris-buffered saline (0.5% tween), membranes were incubated with secondary HRP-conjugated antibody (1:2500, (mouse, cat# 31,430) and (rabbit, cat# 31,460); Pierce Thermo) for 1 h followed by 1 min. incubation with SuperSignal Pico (Pierce, Thermo) and imaging with ChemiDoc (Bio-Rad).

Uncropped blots, including size markers, are shown in Supplementary Fig. 9.

### Sequencing and peak calling

Sequence reads (50-mer) were generated for ChIP-seq and RNA-seq experiments on the Illumina HiSeq 2000 and Illumina NextSeq 500 platforms at the Advanced Technology Center (ATC), National Cancer Institute (NCI) (Rockville, MD, USA) and the tags were uniquely aligned to the mouse reference genome (NCBI37/mm9 assembly). Regions of enriched tags (termed ‘peaks’, TF binding sites or H3K27ac regions) were called using MACS2 with default parameters (‘broadPeak for H3K27ac and ‘narrowPeak’ for STAT3)

### Tag density profiles (used for genome browser screen shots)

We constructed a tag density profile of the data by extending each mapped read to the 150 bp length into the 3′ direction relative to that strand and counted the distribution of tag counts over the genome. The scale factor is given by 10 million per the total number of non-mitochondrial reads. By multiplying the scale factor, the normalized tag density profiles were obtained.

### Differential ChIP sites and motif analyses

Differentially regulated H3K27ac or STAT3 sites were identified from three (H3K27ac) or two (STAT3) biological replicates using DESeq^65^ in the default parameters through HOMER^66^ (http://homer.salk.edu/homer/). Cutoffs: fold change ≥2, adjusted *p*-value ≤0.1, measured using DESeq (Supplementary Data 4).

*De-novo* motif analysis (all enriched motifs are presented, *p*-value ≤1^−10^, binomial), motif proximities to binding sites and STAT3 motif score performed using HOMER.

### Box plots, aggregation plots and heatmaps

Box plots represent sequenced tag density (per bp) around (±100 bp) the center of binding sites or as detailed in legend. The median is denoted by a horizontal line and the mean is denoted by a ‘+’ sign. Aggregation plots represent average sequenced tag density (10 bp bins) around (±500 bp or as detailed in legend) the center of binding sites and H3K27ac sites. Heatmaps show STAT3 ChIP tag density centered on STAT3 peaks (±4 kb). All plots generated using HOMER.

### Statistics

Asterisks denote statistical significance as determined by an unpaired, two-tailed t-test. Single asterisk denotes *p*-value ≤ 0.05, double asterisks denote *p*-value ≤ 0.01, NS denote not significant. All RNA-seq and ChIP-seq *p*-values were adjusted for multiple comparisons. Statistical tests used in the study are commonly used and considered appropriate for the hypotheses tested. The data meet assumptions of population distribution. Variance between the groups that are being statistically compared is similar.

### Published data sets

H3K4me2 ChIP-seq (GEO accession# GSE65167)^67^, p300 and CBP ChIP-seq (ArrayExpress accession# E-MTAB-941)^68^, DNase-seq (GEO accession# GSE72087)^37^ GSEA datasets GEO accession#: LPS-GSE37546^69^, pneumonia-GSE35516^9^, NASH-GSE63067^70^, HCV-GSE51699^71^.

### Study approval

All animal procedures were approved by the Animal Users and Care Committee, the National Cancer Institute, National Institutes of Health.

### Data availability

All high throughput sequencing data are summarized in Supplementary Data 6 and have been submitted to the NCBI Gene Expression Omnibus under accession number GSE96770.

## Electronic supplementary material

Supplementary Information

Description of Additional Supplementary Files

Supplementary Data 1

Supplementary Data 2

Supplementary Data 3

Supplementary Data 4

Supplementary Data 5

Supplementary Data 6
