# Supplementary material for: Synergistic gene expression during the acute phase response is characterized by transcription factor assisted loading
Source: Nat Commun. 2017 Nov 29;8:1849. doi: 10.1038/s41467-017-02055-5 (PMC5707366; doi:10.1038/s41467-017-02055-5)
Supplement: Supplementary file 1 — Supplementary Information [file 41467_2017_2055_MOESM1_ESM.pdf]

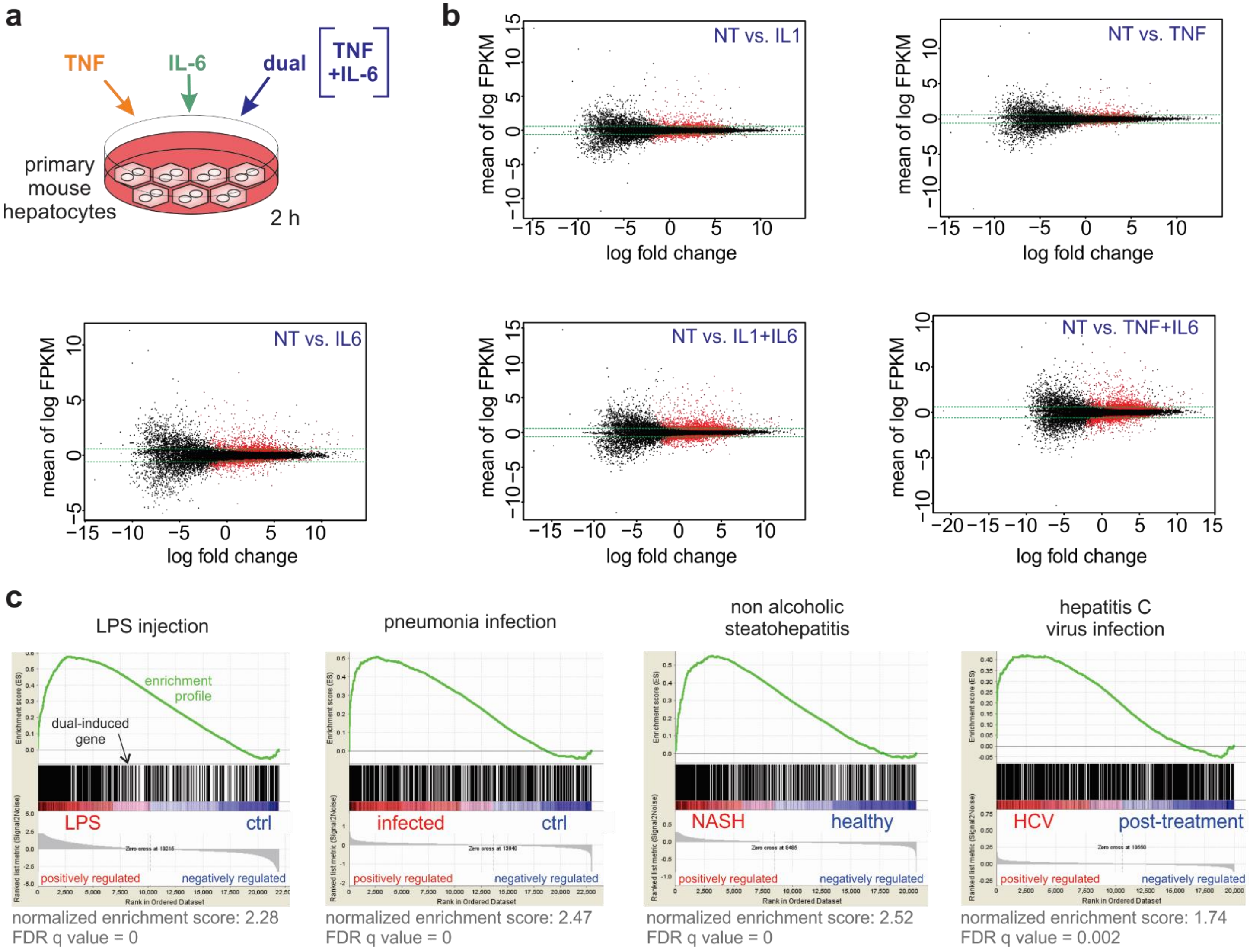

Supplementary Figure 1: Pro-inflammatory cytokine treatments profoundly affect hepatic transcriptome in a manner reminiscent of *in vivo* acute and chronic inflammation.

(a) Experimental setup used for TNF-IL-6 crosstalk experiments. Primary mouse hepatocytes were treated with TNF (2 ng ml^{-1}), IL-6 (10 ng ml^{-1}) or both for 2 h. Then, cells were collected for downstream experiments.

(b) MA plots depicting global gene expression changes following cytokine treatment. Red dots represent genes significantly changing in expression in the indicated treatment compared to the non-treated control (adjusted p value ≤ 0.05, measured with CuffDiff). Green lines mark the 1.5 fold change cutoff (FPKM = fragments per kb per million reads).

(c) Gene set enrichment analysis (GSEA) of dual-induced genes with respect to ranked genes from four different datasets: LPS-injected mice[1], *Streptococcus pneumoniae*-infected mice[2] and livers from NASH[3] or HCV[4] patients. Vertical black lines in the barcode-like appearance represent dual-induced genes spread along the reference dataset. The left-hand side of the x axis shows the most induced genes in the reference dataset. Bright green line shows the enrichment trend. In all datasets, dual-induced genes are enriched in the inflammatory state compared to control (GSEA q value and normalized enrichment score are detailed in the bottom of each plot).

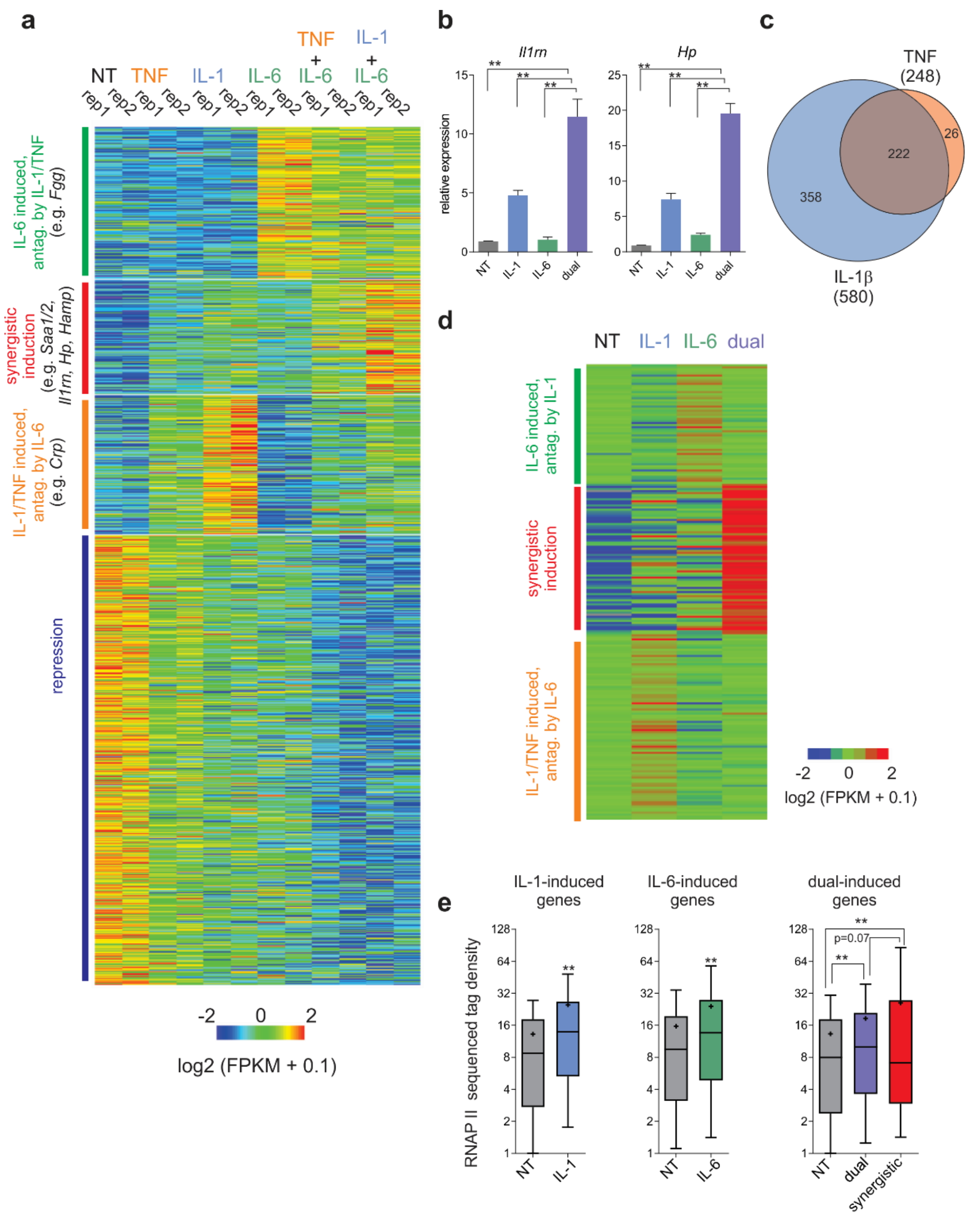

Supplementary Figure 2: A multifaceted response of hepatocytes to pro-inflammatory cytokines.

(a) K-means clustering (k = 4) of cytokine-regulated genes (n = 3,260) reveals a multifaceted gene-induction program following pro-inflammatory cytokine treatment. The expression of a given gene induced by one cytokine can be antagonized or synergized by the second cytokine (see left-hand side for examples).

(b) Nascent RNA levels of synergistic genes in primary hepatocytes following a 2 h cytokine treatment. Representative experiment shown of at least three independent repeats. Error bars represent s.d. of three technical replicates.

(c) Venn diagram portraying the extensive overlap between IL-1β- and TNF-induced genes as well as the higher number of genes induced by IL-1β (in parentheses is the total number of genes induced under each condition).

(d) K-means clustering (k = 3) of antagonistic and synergistic genes that met cutoffs specified in the main text (n = 221).

(e) RNAP II increases in a cytokine-dependent manner at cytokine-induced genes (RNAP II ChIP-seq tag density measured across first 200 bp downstream of transcription start site – TSS).

\- Double asterisks denote p value ≤ 0.01 as determined by an unpaired, two-tailed t-test.

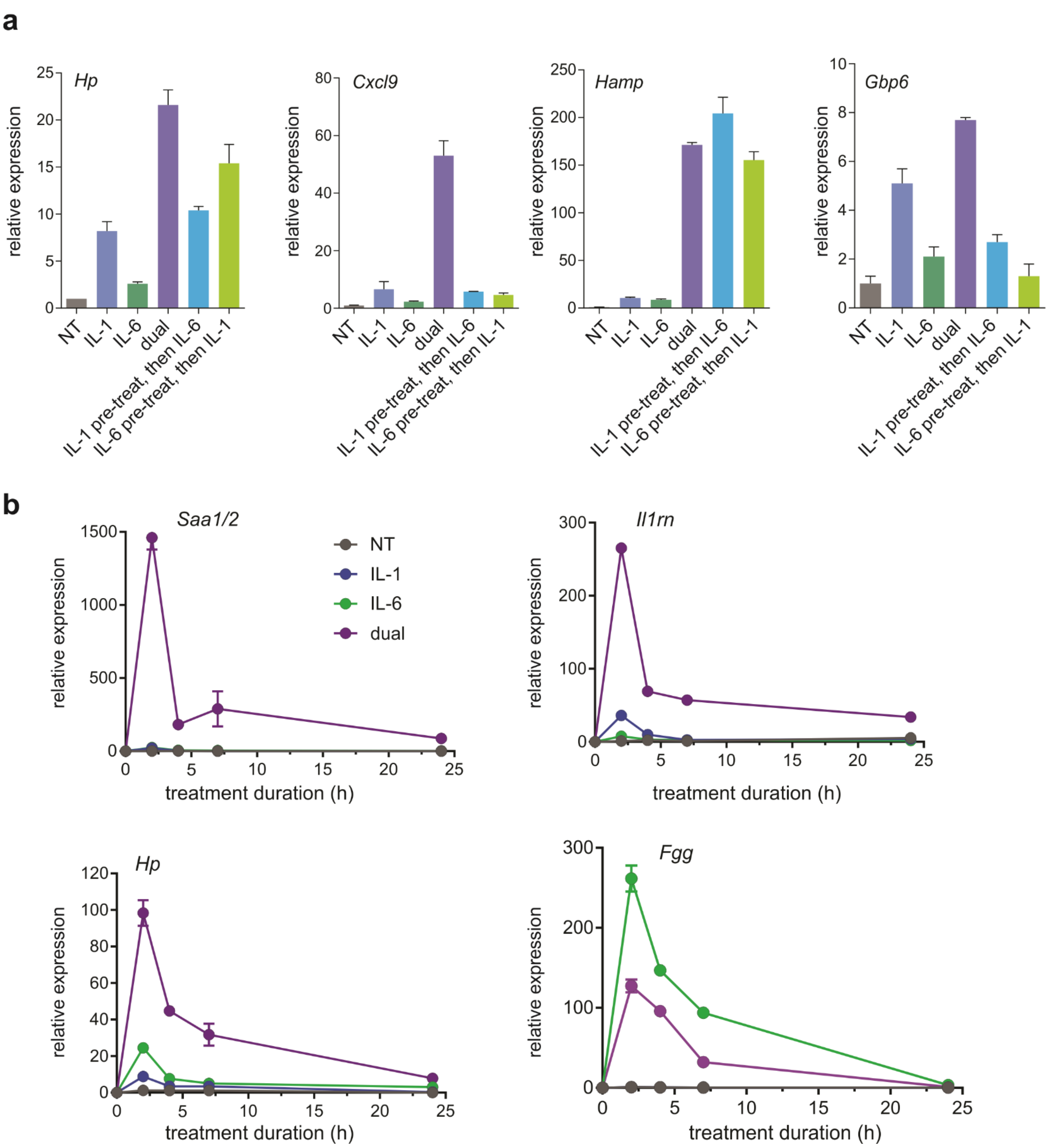

Supplementary Figure 3: Cytokine-induced genes are regulated immediately following signal.

(a) Nascent RNA levels of synergistic genes in primary hepatocytes sequentially treated with either IL-1β or IL-6 for 6 h followed by a 2 h treatment with the second cytokine. Compare to simultaneous 2 h treatment (dual).

(b) Nascent RNA levels of synergistic and antagonistic genes in primary hepatocytes following cytokine treatment spanning 2-24 h. Patterns show that both synergistic and antagonistic expression is brought about immediately and only decreases with time.

\- Representative experiments shown of at least three independent repeats. Error bars represent s.d. of three technical replicates.

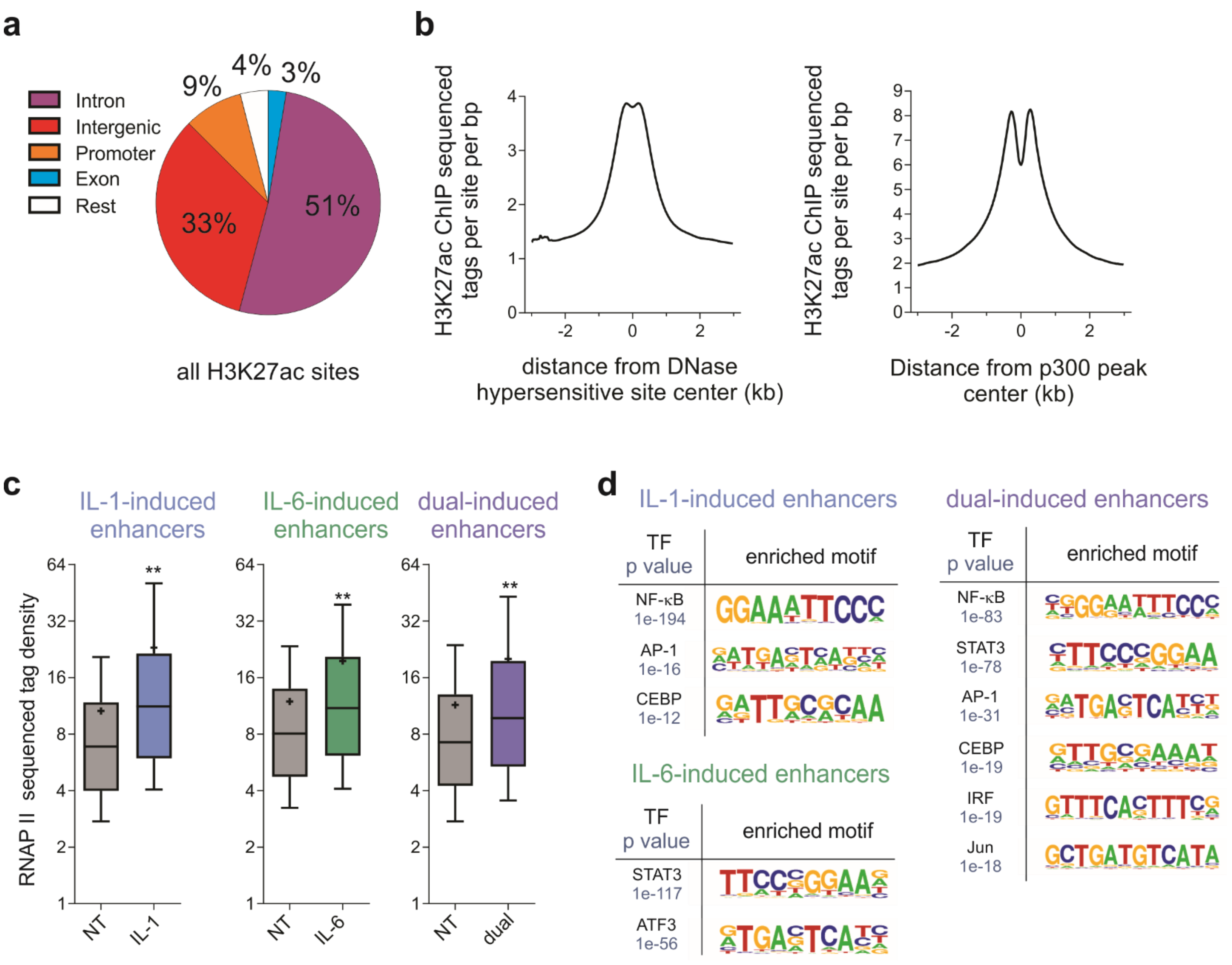

Supplementary Figure 4: Properties of cytokine-induced enhancers.

(a) Genomic annotation of all enriched H3K27ac regions (these regions were classified as enhancers for further analyses).

(b) H3K27ac ChIP-seq tag density in the vicinity of two enhancer marks: liver DNase hypersensitive sites (adopted from Goldstein et al.[5]) and p300 binding sites (adopted from Faure et al.[6]).

(c) RNAP II increases in a cytokine-dependent manner at cytokine-induced enhancers (RNAP II ChIP-seq tag density measured at +/- 500 bp from center of enhancer). Double asterisks denote p value ≤ 0.01 as determined by an unpaired, two-tailed t-test.

(d) Enriched TF motifs in cytokine-induced enhancers (all enriched motifs are shown).

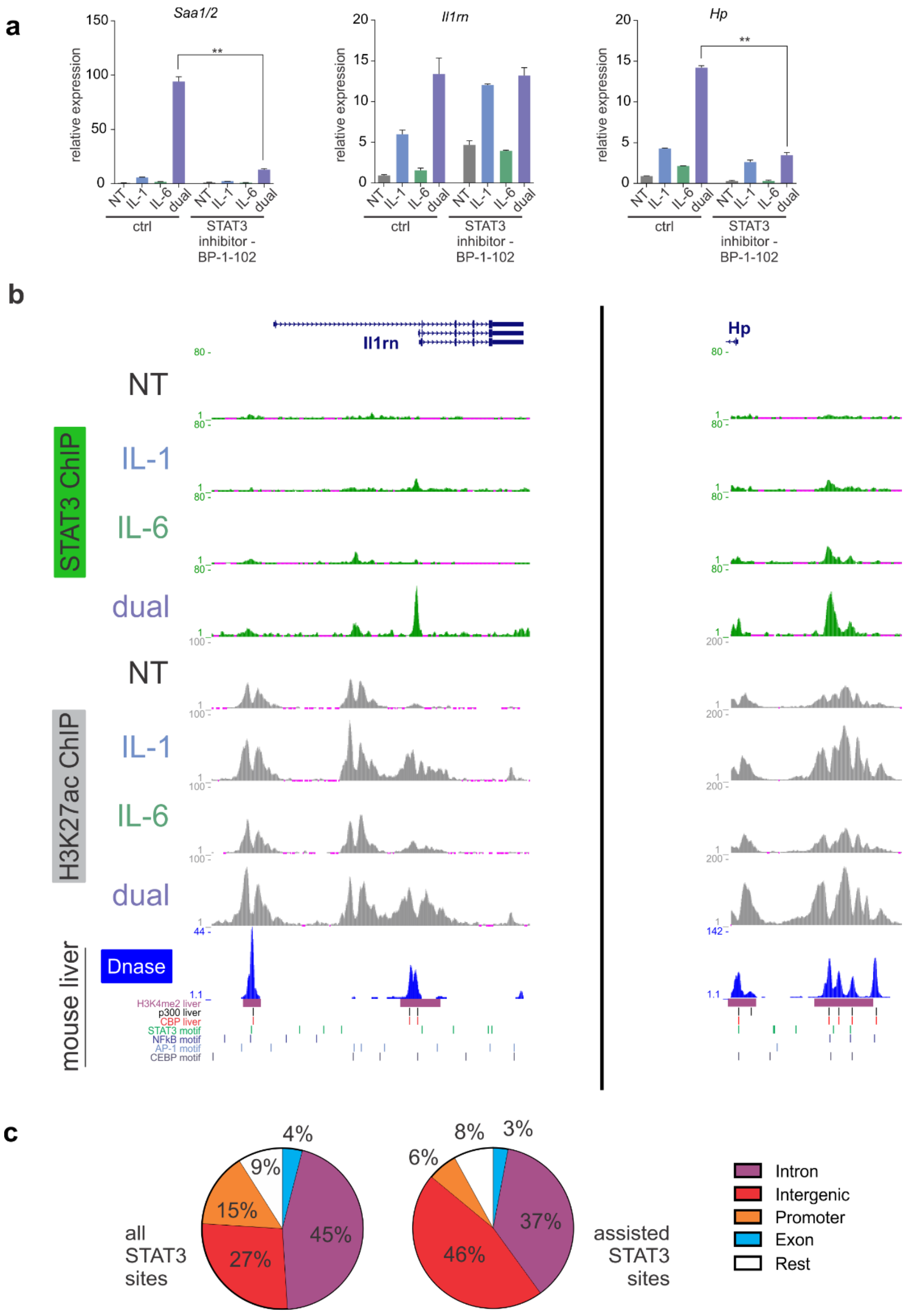

Supplementary Figure 5: STAT3 is essential for synergistic gene expression and its binding is augmented by IL-1β at enhancers proximal to synergistic genes.

(a) Nascent RNA levels of synergistic genes in primary hepatocytes pre-treated with a STAT3 inhibitor followed by cytokine treatments. Representative experiment shown of at least three independent repeats. Error bars represent s.d. of three technical replicates. Double asterisks denote p value ≤ 0.01 as determined by an unpaired, two-tailed t-test.

(b) Genome browser tracks showing IL-1β-dependent activation of enhancers proximal to synergistic genes and the dependence on IL-1β for maximal STAT3 binding.

(c) Genomic annotation of all STAT3 sites (left) and only assisted sites (right).

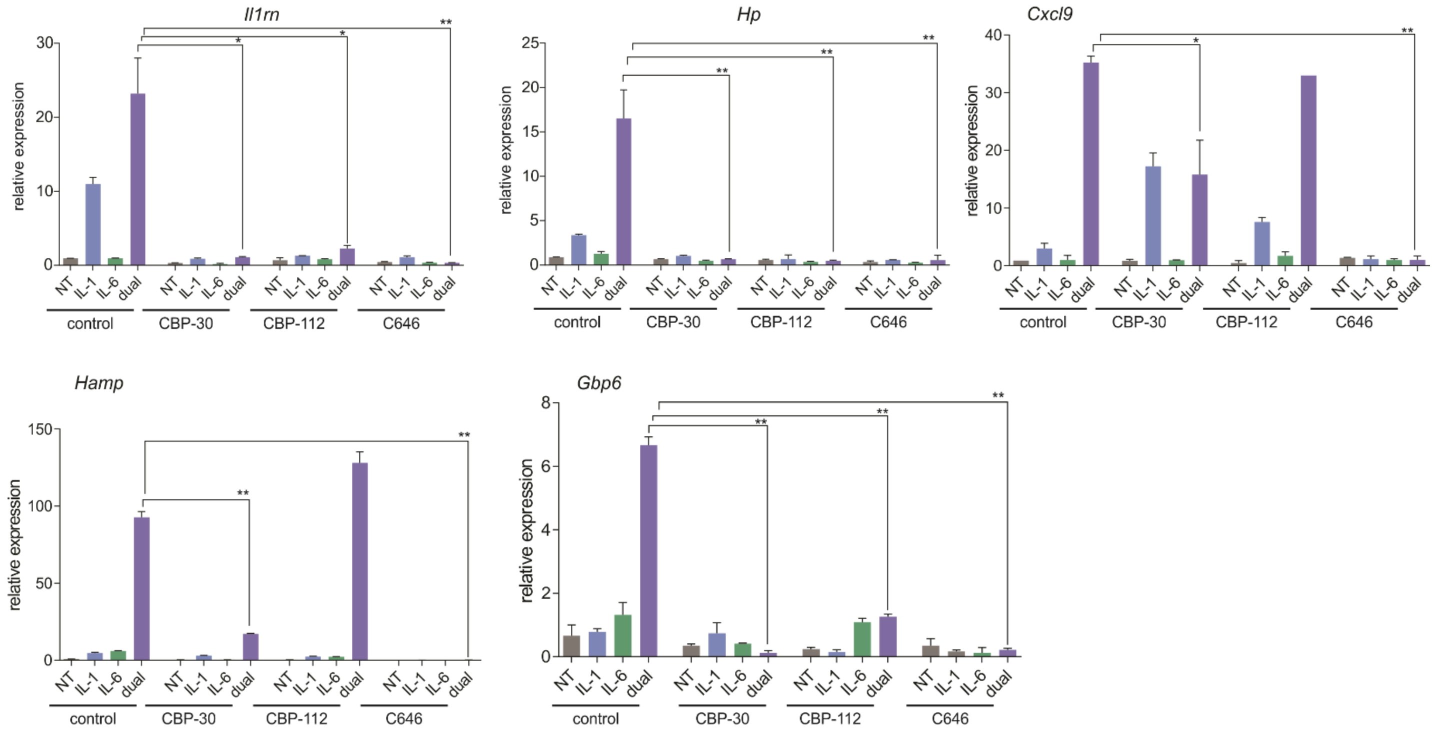

Supplementary Figure 6: Synergistic gene expression is p300/CBP-dependent.
Nascent RNA levels of synergistic genes in primary hepatocytes pre-treated with p300/CBP inhibitors followed by indicated cytokine treatments. Representative experiment shown of at least three independent repeats. Error bars represent s.d. of three technical replicates. Asterisks denote statistical significance as determined by an unpaired, two-tailed t-test. Single asterisk denotes p value ≤ 0.05, double asterisks denote p value ≤ 0.01.

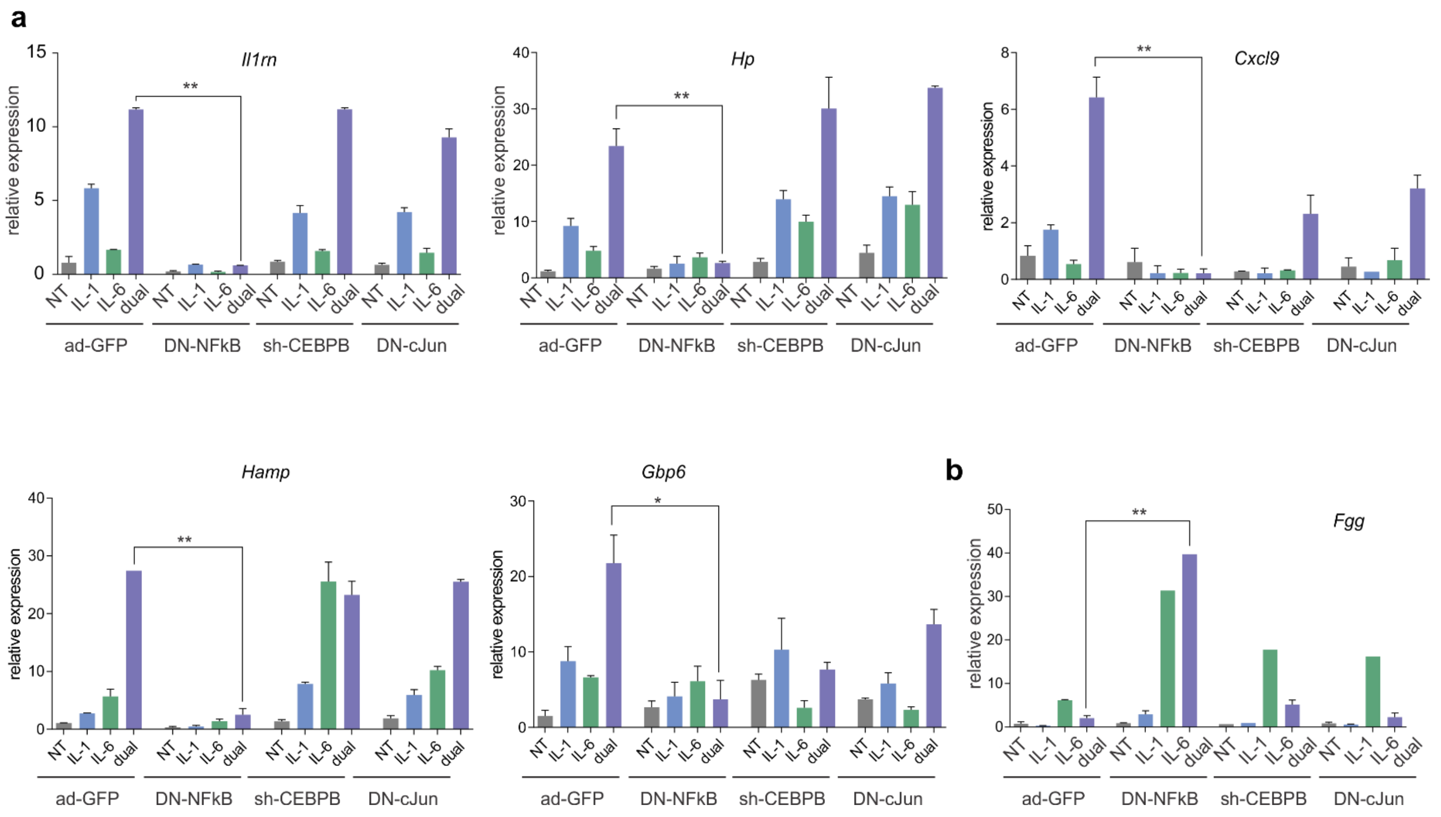

Supplementary Figure 7: NF-κB is essential for synergistic gene expression.

(a) Nascent RNA levels of synergistic genes in primary hepatocytes pre-treated with adenoviral inhibitors of IL-1β-activated TFs.

(b) Nascent RNA levels of *Fgg* (an IL-1β-antagonized, IL-6-induced gene) in primary hepatocytes pre-treated with adenoviral inhibitors of IL-1β-activated TFs.

- Representative experiments shown of at least three independent repeats. Error bars represent s.d. of three technical replicates. Asterisks denote statistical significance as determined by an unpaired, two-tailed t-test. Single asterisk denotes p value ≤ 0.05, double asterisks denote p value ≤ 0.01.

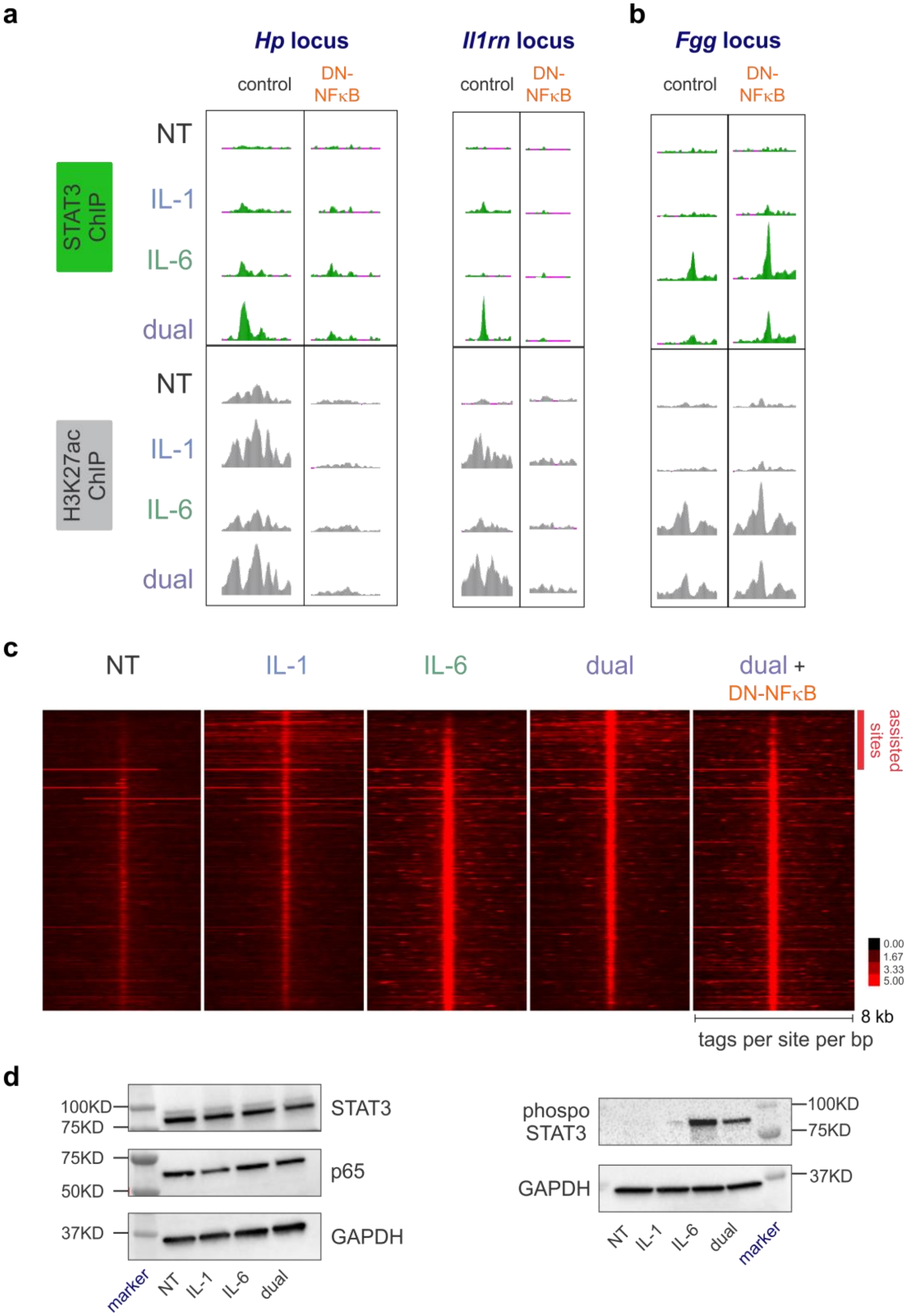

Supplementary Figure 8: NF-κB is essential for STAT3 assisted loading.

(a) STAT3 assisted loading as well as enhancer priming at the *Hp* and *Il1rn* loci are negated in the presence of a dominant negative peptide inhibiting NF-κB activity (DN-NFκB).

(b) A dominant negative peptide inhibiting NF-κB activity does not curtail, but rather increases STAT3 binding and H3K27ac at the *Fgg* locus (next to *Fgg*, an IL-1β-antagonized, IL-6-induced gene).

(c) Heat map depicting intensity of STAT3 binding sites sorted by fold change (dual over IL-6).

(d) Western blot analysis of p65, STAT3 and phospho-STAT3 following 2 h of cytokine treatments.

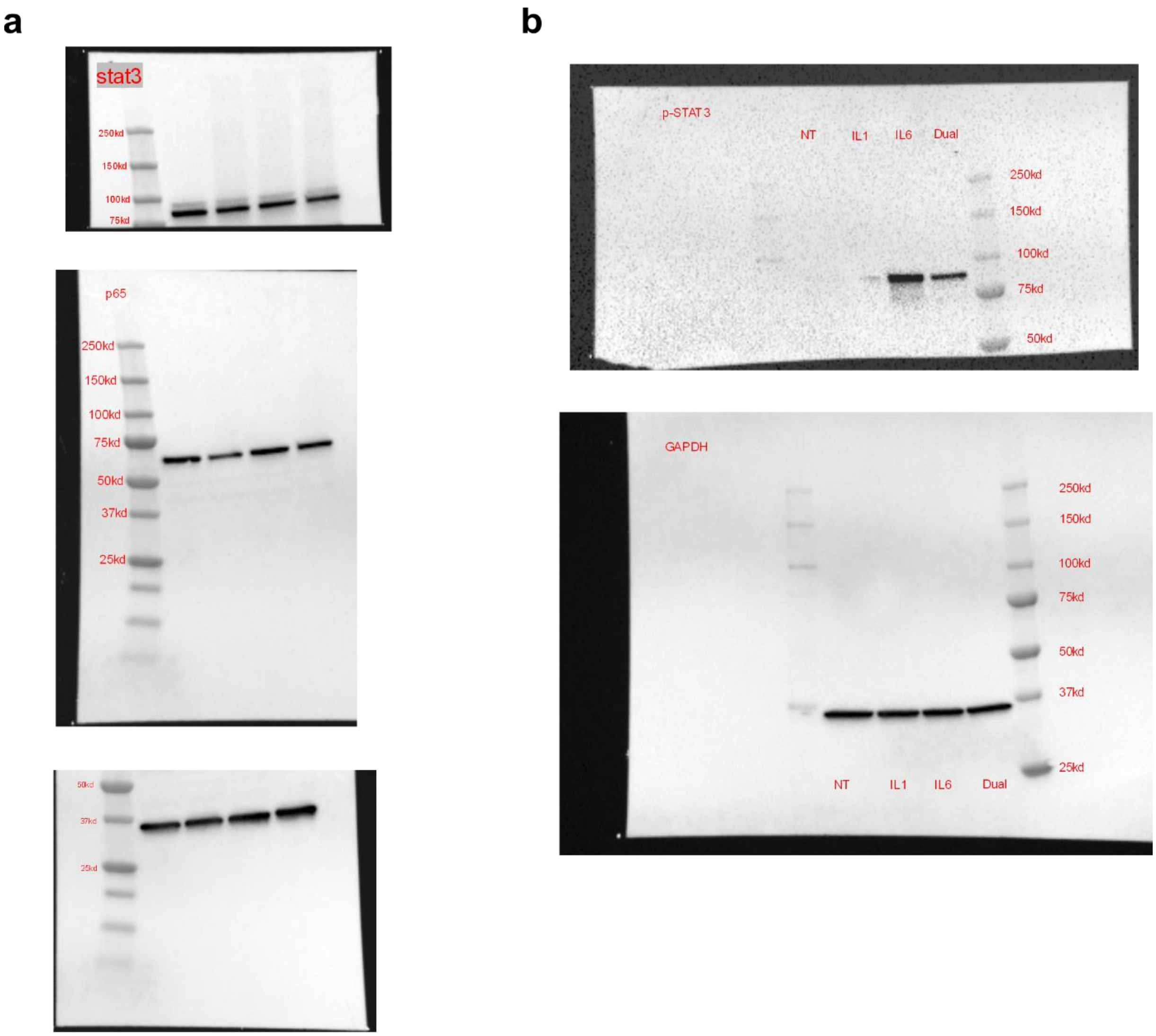

Supplementary Figure 9: uncropped western blots.
Western blot analysis of p65, STAT3, phospho-STAT3 and GAPDH following 2 h of cytokine treatments. Panel a corresponds to the left-hand side of Supplementary Fig. 8d. Panel b corresponds to the right-hand side of Supplementary Fig. 8d.

Supplementary References

1. Herrema, H. et al. Disturbed hepatic carbohydrate management during high metabolic demand in medium-chain acyl-CoA dehydrogenase (MCAD)-deficient mice. *Hepatology* **47**, 1894-904 (2008).
2. Quinton, L.J. et al. Hepatocyte-specific mutation of both NF-kappaB RelA and STAT3 abrogates the acute phase response in mice. *J Clin Invest* **122**, 1758-63 (2012).
3. Frades, I. et al. Integrative genomic signatures of hepatocellular carcinoma derived from nonalcoholic Fatty liver disease. *PLoS One* **10**, e0124544 (2015).
4. Meissner, E.G. et al. Endogenous intrahepatic IFNs and association with IFN-free HCV treatment outcome. *J Clin Invest* **124**, 3352-63 (2014).
5. Goldstein, I. et al. Transcription factor assisted loading and enhancer dynamics dictate the hepatic fasting response. *Genome Res* **27**, 427-439 (2017).
6. Faure, A.J. et al. Cohesin regulates tissue-specific expression by stabilizing highly occupied cis-regulatory modules. *Genome Res* **22**, 2163-75 (2012).
