# Supplementary material for: Synergistic gene expression during the acute phase response is characterized by transcription factor assisted loading
Source: Nat Commun. 2017 Nov 29;8:1849. doi: 10.1038/s41467-017-02055-5 (PMC5707366; doi:10.1038/s41467-017-02055-5)
Supplement: Supplementary file 2 — Description of Additional Supplementary Files [file 41467_2017_2055_MOESM2_ESM.pdf]

Description of Additional Supplementary Files

File Name: Supplementary Data 1
Description: All cytokine-regulated genes, k-means clustered (k = 4).

File Name: Supplementary Data 2
Description: All cytokine-induced genes, by groups (related to Fig. 1d).

File Name: Supplementary Data 3
Description: STAT3 binding sites called in each condition.

File Name: Supplementary Data 4
Description: Differentially regulated H3K27ac and STAT3 sites.

File Name: Supplementary Data 5
Description: List of the nearest gene to every assisted site.

File Name: Supplementary Data 6
Description: Details of all RNA-seq and ChIP-seq samples.
